# Increased Aurora B expression reduces substrate phosphorylation and induces chromosomal instability

**DOI:** 10.3389/fcell.2022.1018161

**Published:** 2022-10-13

**Authors:** Eric M. C. Britigan, Jun Wan, Daniel K. Sam, Sarah E. Copeland, Amber L. Lasek, Laura C. F. Hrycyniak, Lei Wang, Anjon Audhya, Mark E. Burkard, Avtar Roopra, Beth A. Weaver

**Affiliations:** ^1^ Molecular and Cellular Pharmacology Graduate Training Program, University of Wisconsin-Madison, Madison, WI, United States; ^2^ Physiology Graduate Training Program, University of Wisconsin-Madison, Madison, WI, United States; ^3^ Cellular and Molecular Biology Graduate Training Program, University of Wisconsin-Madison, Madison, WI, United States; ^4^ Department of Biomolecular Chemistry, University of Wisconsin-Madison, Madison, WI, United States; ^5^ Carbone Cancer Center, University of Wisconsin-Madison, Madison, WI, United States; ^6^ Department of Neuroscience, University of Wisconsin-Madison, Madison, WI, United States; ^7^ Department of Medicine, University of Wisconsin-Madison, Madison, WI, United States; ^8^ Department of Oncology/McArdle Laboratory for Cancer Research, University of Wisconsin-Madison, Madison, WI, United States; ^9^ Department of Cell and Regenerative Biology, University of Wisconsin-Madison, Madison, WI, United States

**Keywords:** mitosis, CIN, spindle assembly checkpoint, mitotic checkpoint, aurora kinase inhibitor

## Abstract

Increased Aurora B protein expression, which is common in cancers, is expected to increase Aurora B kinase activity, yielding elevated phosphorylation of Aurora B substrates. In contrast, here we show that elevated expression of Aurora B reduces phosphorylation of six different Aurora B substrates across three species and causes defects consistent with Aurora B inhibition. Complexes of Aurora B and its binding partner INCENP autophosphorylate in trans to achieve full Aurora B activation. Increased expression of Aurora B mislocalizes INCENP, reducing the local concentration of Aurora B:INCENP complexes at the inner centromere/kinetochore. Co-expression of INCENP rescues Aurora B kinase activity and mitotic defects caused by elevated Aurora B. However, INCENP expression is not elevated in concert with Aurora B in breast cancer, and increased expression of Aurora B causes resistance rather than hypersensitivity to Aurora B inhibitors. Thus, increased Aurora B expression reduces, rather than increases, Aurora B kinase activity.

## Introduction

Aurora B is a serine/threonine kinase essential for accurate completion of multiple steps during mitosis and cytokinesis. Of its multiple binding partners, (Kim et al., 1999; Adams et al., 2000; Speliotes et al., 2000; Uren et al., 2000; Gassmann et al., 2004; Carmena et al., 2012; Capalbo et al., 2019), INCENP specifically stimulates Aurora B kinase activity *in vitro* (Honda et al., 2003). INCENP binding partially activates Aurora B, resulting in phosphorylation of other Aurora B:INCENP complexes in trans at T232 on Aurora B and on the TSS motif of INCENP (Bishop and Schumacher, 2002; Honda et al., 2003; Sessa et al., 2005; Rosasco-Nitcher et al., 2008). This results in a conformational change in Aurora B, bestowing full kinase activity (Sessa et al., 2005). Because autophosphorylation occurs in trans, Aurora B activity is proportional to the local concentration of Aurora B: INCENP complexes, and increasing the local concentration increases Aurora B activity in *Xenopus* extracts and human cells (Kelly et al., 2007; Wang et al., 2011).

Aurora B is a chromosomal passenger protein which exhibits a dynamic localization throughout mitosis (Carmena et al., 2012). During early stages of mitosis, Aurora B localizes to centromeres and kinetochores, protein based structures that assemble on the centromeric region of DNA and serve as linkers between chromosomes and spindle microtubules (Cleveland et al., 2003; DeLuca et al., 2011; Broad et al., 2020; Marsoner et al., 2022). Aurora B then transitions to the spindle midzone in anaphase and the midbody during telophase, where it functions in cytokinesis (Cooke et al., 1987; Douglas et al., 2010). Early in mitosis, Aurora B plays a universally accepted role in ensuring proper biorientation by releasing microtubule attachments that are not under tension, which permits accurate tension-generating (amphitelic) attachments to be formed (Biggins et al., 1999; Tanaka et al., 2002; Cimini et al., 2006; Pinsky et al., 2006). This error correcting feature of Aurora B generates unattached kinetochores that activate the mitotic spindle assembly checkpoint, the major cell cycle checkpoint acting during mitosis, which delays mitotic progression until all kinetochores are stably attached to spindle microtubules. Whether Aurora B functions directly in mitotic checkpoint signaling, independent of its role in error correction, has been controversial. Early work reported that Aurora B was necessary to activate the mitotic checkpoint in response to lack of tension caused by taxol, but not in response to unattached kinetochores caused by loss of microtubules (Biggins et al., 1999; Biggins and Murray, 2001; Carvalho et al., 2003; Pinsky et al., 2006). However, others have reported that Aurora B also functions directly in mitotic checkpoint activation, even in the absence of microtubules, and contributes to formation of mitotic checkpoint complexes (Santaguida et al., 2011; Matson et al., 2012; Matson and Stukenberg, 2014; Hadders et al., 2020; Roy et al., 2022).

Consistent with its diverse localization and function, depletion or inhibition of Aurora B causes pleiotropic defects, including misaligned and lagging chromosomes and cytokinesis failure (Kaitna et al., 2000; Ditchfield et al., 2003; Hauf et al., 2003; Santaguida et al., 2011; Carmena et al., 2012; Park et al., 2018). These types of defects are common in tumors. However, Aurora B mutation, deletion, and underexpression are relatively infrequent, occurring in ≤6% of cancers (Cerami et al., 2012; Gao et al., 2013). However, Aurora B is commonly overexpressed in tumors, including those of the lung (Smith et al., 2005; Vischioni et al., 2006; Takeshita et al., 2013), breast (Zhang et al., 2015; Liu et al., 2022), colon (Bischoff et al., 1998; Katayama et al., 1999; Tuncel et al., 2012), and bone (Wu et al., 2020) where it often serves as a marker of poor prognosis. At the mRNA level, Aurora B exhibits increased expression relative to normal samples in ∼45% of stomach cancers, ∼60% of prostate and colorectal cancers, 75% of head and neck cancers, and ≥80% of cholangiocarcinoma, bladder, breast, esophageal, kidney, liver, lung, and uterine cancers (Cerami et al., 2012; Gao et al., 2013). Since Aurora B is a cell cycle regulated gene (Tatsuka et al., 1998; Terada et al., 1998; Kimura et al., 2004), it is unclear to what extent increased Aurora B expression is simply a marker of the increased proliferative rate of tumors. However, >90% of mice that overexpress Aurora B develop tumors, confirming that Aurora B overexpression can be sufficient for transformation (González-Loyola et al., 2015). It is expected that tumors with elevated levels of Aurora B are addicted to oncogenic signaling from the increased kinase activity of Aurora B, and several Aurora B kinase inhibitors have entered clinical trials (Sharma and Settleman, 2007; Altieri, 2008; Boss et al., 2009; Azzariti et al., 2011; Kantarjian et al., 2013a; Foran et al., 2014; Geuns-Meyer et al., 2015; Schöffski et al., 2015).

The consequences of experimentally elevating Aurora B expression have previously been unclear. Reports agree that expression of kinase dead Aurora B causes multinucleation, presumably due to cytokinesis defects (Katayama et al., 1998; Tatsuka et al., 1998; Terada et al., 1998; Ota et al., 2002; Honda et al., 2003; Girdler et al., 2006). Expression of wild type Aurora B, typically used as a control for kinase dead Aurora B, was observed to induce low levels of multinucleation, substantially lower than the level induced by expression of kinase dead Aurora B, in early studies (Katayama et al., 1998; Terada et al., 1998). However, later studies reported that elevated expression of wild type Aurora B caused no increase in cytokinesis failure, multinuclearity, or polyploidy (Honda et al., 2003; Girdler et al., 2006). In studies that identified multinucleation, the mechanism was inferred to be cytokinesis failure, presumably due to increased Aurora B kinase activity (Ota et al., 2002; Girdler et al., 2006), although premature cohesin dissociation without cytokinesis failure was also proposed as a potential mechanism of tetraploidization (Nguyen et al., 2009). Reports on the effects of elevated wild type Aurora B expression on nuclear division have also been conflicting, with one study concluding that elevated wild type Aurora B causes an increase in lagging and bridge chromosomes (Ota et al., 2002) while others concluded that increased expression of wild type Aurora B does not cause defects in chromosome congression or segregation during mitosis (Terada et al., 1998; Murata-Hori et al., 2002). Thus, the consequences of elevated expression of wild type Aurora B have remained unclear.

Here we show that increased Aurora B expression results in reduced phosphorylation of Aurora B substrates in worm, murine and human cells. Consistent with this decrease in substrate phosphorylation, increased expression of Aurora B produces mitotic and cytokinetic defects identical to those caused by depletion or inhibition of Aurora B, including misaligned and lagging chromosomes, and cytokinesis failure (Kaitna et al., 2000; Ditchfield et al., 2003; Hauf et al., 2003; Carmena et al., 2012). Aurora B upregulation also causes mitotic checkpoint defects independent of error correction. Increased expression of Aurora B mislocalizes INCENP, and co-expression of INCENP with Aurora B rescues Aurora B substrate phosphorylation and mitotic defects. However, INCENP expression does not correlate with Aurora B expression in human breast cancers, and increased Aurora B expression causes resistance rather than hypersensitivity to Aurora B inhibition. These findings demonstrate that the ratio of Aurora B to INCENP is critical for full Aurora B activity and that increased expression of Aurora B without concomitant expression of INCENP, as is common in breast cancer, results in reduced rather than elevated levels of Aurora B substrate phosphorylation.

## Results

### ARF^−/−^ murine embryonic fibroblasts have elevated levels of Aurora B protein, but reduced levels of Aurora B substrate phosphorylation

**FIGURE 1:**
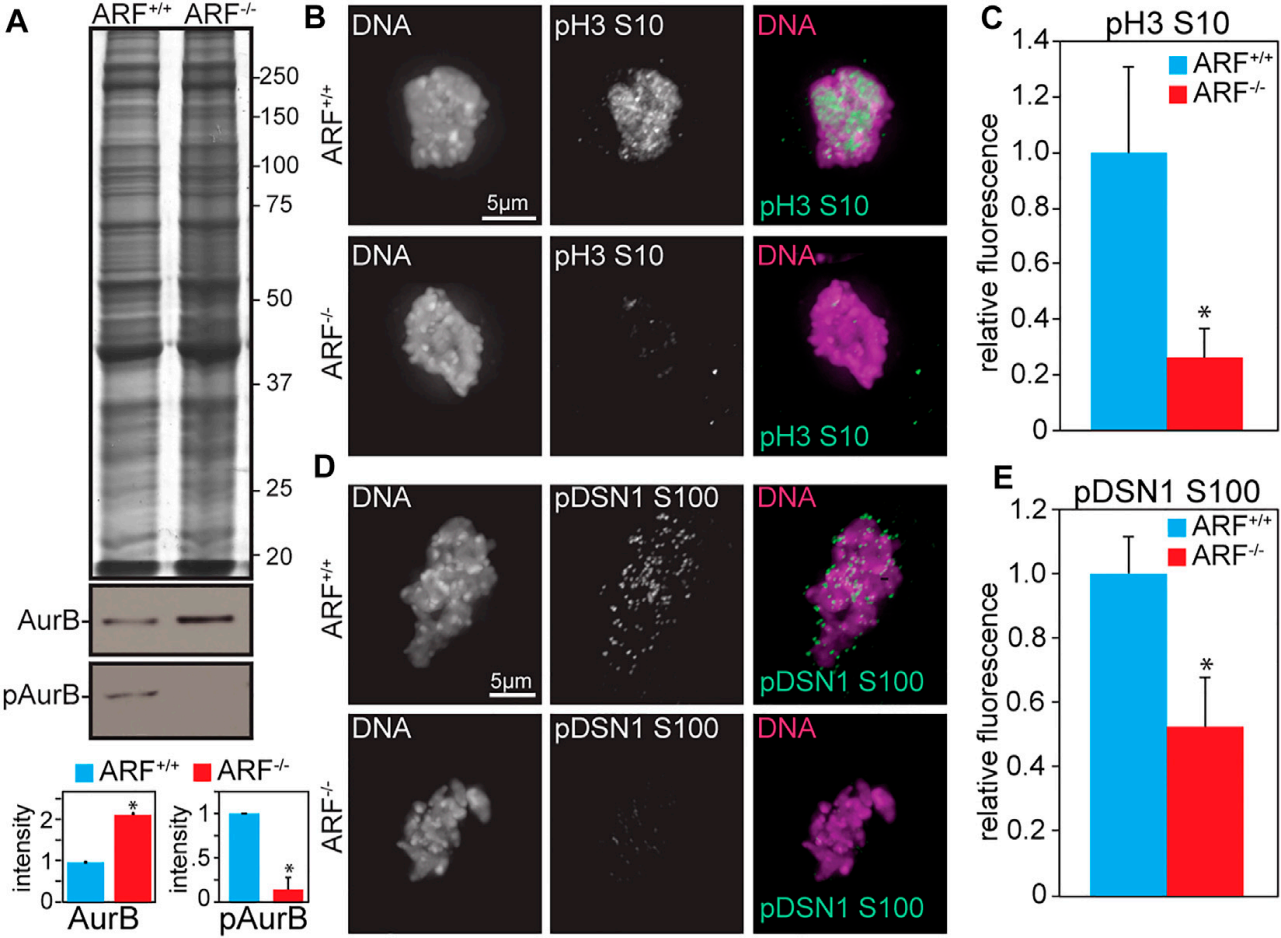
ARF loss results in increased Aurora B protein but reduced Aurora B activity. **(A)** Immunoblot of ARF^+/+^ and ARF^−/−^ MEFs showing Aurora B is increased in ARF^−/−^ MEFs while pT237 (active) Aurora B is decreased. Coomassie is shown as a loading control. Quantitation is shown below. **(B–E)** Quantitative immunofluorescence of pH3 and pDSN1 in ARF^+/+^ and ARF^−/−^ MEFs. **(B,D)** Representative images of pH3 **(B)** and pDSN1 **(D)** in ARF^+/+^ and ARF^−/−^ MEFs. **(C,E)** Graphs showing quantification of pH3 **(C)** and pDSN1 **(D)**. *n* > 10 cells from each of 3 independent experiments. *p < 0.05.

We previously demonstrated that loss of the ARF (Alternative Reading Frame of p16INK4a) tumor suppressor causes misaligned and lagging chromosomes, mitotic checkpoint signaling defects, and cytokinesis failure in primary Murine Embryonic Fibroblasts (MEFs) generated from E14.5 day embryos (Britigan et al., 2014). ARF^−/−^ MEFs express increased protein levels of Aurora B due to reduced protein turnover. Partial knockdown of Aurora B in ARF^−/−^ cells to near-wild type levels rescues their mitotic defects, and exogenous overexpression of Aurora B in wild type MEFs phenocopies ARF loss (Britigan et al., 2014). Because ARF^−/−^ MEFs contain higher levels of Aurora B protein than wild type MEFs, we expected ARF^−/−^ MEFs would have increased Aurora B kinase activity. However, despite the elevated protein levels of Aurora B in ARF^−/−^ MEFs, they contained reduced active Aurora B, as shown by decreased autophosphorylation in the activation loop [T232 in human; T237 in mouse; (Yasui et al., 2004); Figure 1A]. This surprising but reproducible result prompted us to assess phosphorylation of additional bona fide Aurora B substrates, including histone H3 [serine 10, hereafter pH3; (Hsu et al., 2000; Adams et al., 2001; Giet and Glover, 2001; Crosio et al., 2002)] and the KMN complex member DSN1 [serine 100; (Welburn et al., 2010)] using quantitative immunofluorescence. Primary ARF^−/−^ cells had a significantly lower level of pH3 (Figures 1B,C) and pDSN1 (Figures 1D,E) than ARF^+/+^ cells, while levels of Aurora B at centromeres remained unchanged (Supplementary Figure S1).

### Expression of Aurora B-YFP reduces Aurora B substrate phosphorylation in wild type MEFs

**FIGURE 2:**
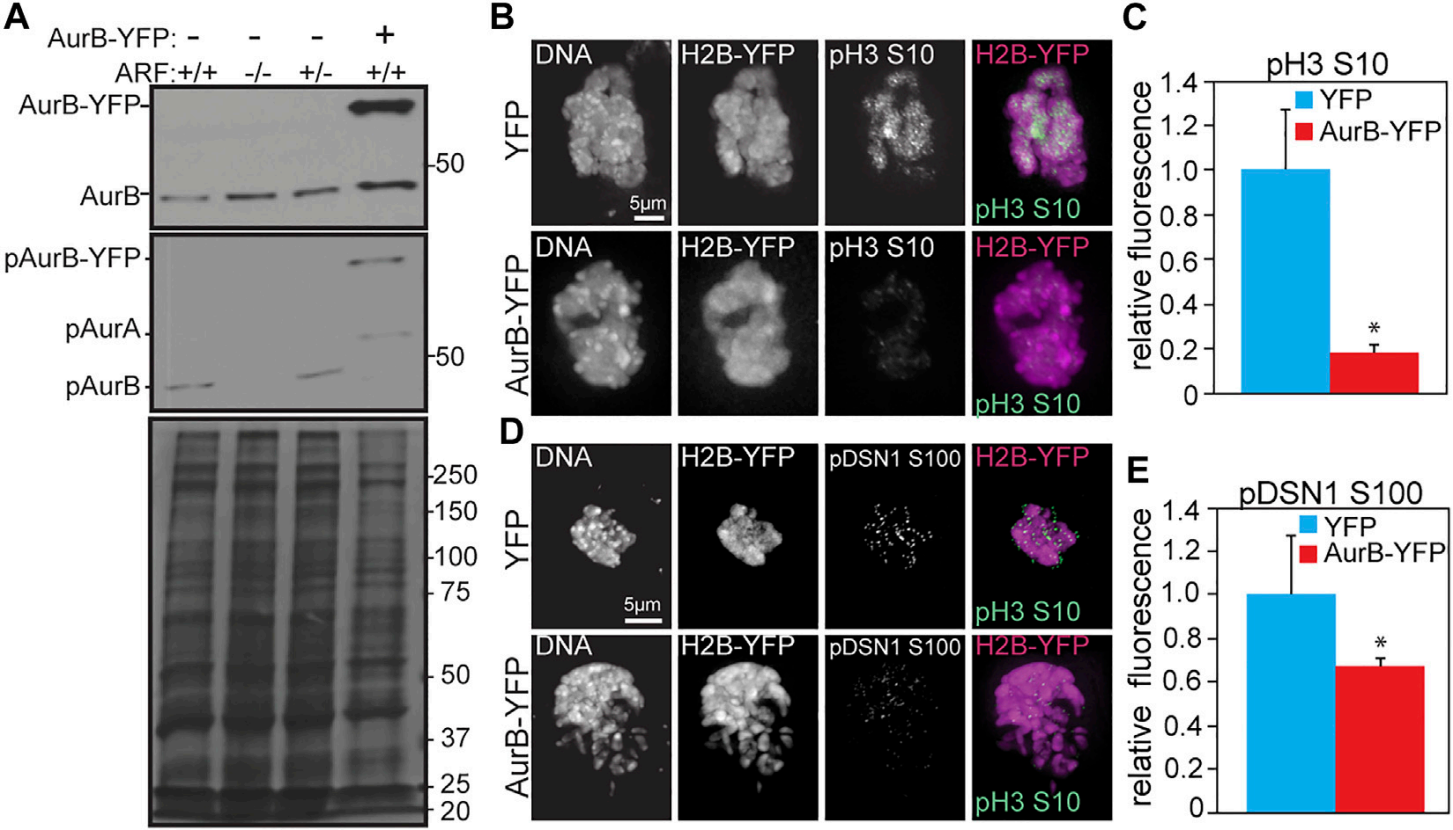
Expression of Aurora B-YFP reduces Aurora B-dependent phosphorylation events in primary MEFs. **(A)** Immunoblot of MEFs of the indicated genotypes±Aurora B-YFP. ARF^−/−^ MEFs show increased Aurora B and decreased pT237 (active) Aurora B. Coomassie (bottom) shown as loading control. **(B–E)** Quantitative immunofluorescence of pH3 and pDSN1. **(B,D)** Representative images of ARF^+/+^ MEFs transfected with YFP or Aurora B-YFP. H2B-YFP was co-transfected and used as a marker of transfected cells. **(C,E)** Graphs showing quantification of phosphoantibody signal in transfected cells. *n* > 10 cells from each of 3 independent experiments. *p < 0.05.

To determine whether Aurora B upregulation or ARF loss was responsible for reduced Aurora B activity in ARF^−/−^ cells, we expressed YFP-tagged Aurora B in wild type MEFs (Figure 2A). We previously showed that expression of Aurora B-YFP in wild type MEFs causes the same defects as Aurora B inhibition or depletion (Britigan et al., 2014), namely misaligned and lagging chromosomes, cytokinesis failure and mitotic checkpoint defects (Ditchfield et al., 2003; Hauf et al., 2003). Examination of pH3 (S10) and pDSN1 (S100) by quantitative immunofluorescence revealed that expression of murine Aurora B with a C-terminal YFP tag in wild type MEFs caused a reduction in phosphorylation of pH3 and pDSN1 similar to that previously observed in ARF^−/−^ cells (Figures 2B–E). The validity of the murine Aurora B cDNA was verified by sequencing (Supplementary Figure S2). Together, these data show that increased expression of Aurora B results in reduced Aurora B substrate phosphorylation in primary MEFs.

### Elevated expression of the Aurora B homolog in *C. elegans* reduces Aurora B substrate phosphorylation and causes mitotic defects

Aurora B function is conserved throughout evolution from yeast to humans. As an initial test of whether increased expression of Aurora B inhibits its activity in other species, pH3 (S10) was measured in the proximal most oocytes of *C. elegans* expressing a GFP-tagged version of the Aurora B worm homologue, AIR-2. Consistent with data in MEFs, GFP-AIR-2 expressing oocytes had reduced pH3 as compared to control oocytes (Supplementary Figures S3A,B). GFP-AIR-2 expression was also associated with lagging chromosomes in the first mitotic division (Supplementary Figure S3C).

### Expression of GFP-Aurora B in HeLa cells reduces Aurora B substrate phosphorylation and causes mitotic defects consistent with Aurora B inhibition

To assess whether increased expression of Aurora B also reduces its substrate phosphorylation in human cells, we created a GFP-tagged Aurora B retroviral construct. We began by verifying the human Aurora B cDNA construct by aligning the predicted protein sequence with four other publicly available sequences (Accession numbers BC080581.1, BC000442.2, BC009751.2, and AB519677.1). The four publicly available sequences showed a single polymorphism. BC009751.2, and AB519677.1 have a threonine at residue 298, while BC080581.1, and BC000442.2 have a methionine (Supplementary Figure S4A). The sequence used here has threonine at position 298. To determine whether this residue is important functionally, we compared the region around residue 298 in six different species (Supplementary Figure S4B). These six species contain five different amino acids at this position, and four species contain nonphosphorylatable residues at this position (Supplementary Figure S4B). We conclude that this residue is not evolutionarily conserved and is unlikely to be an important site of phosphorylation.

**FIGURE 3:**
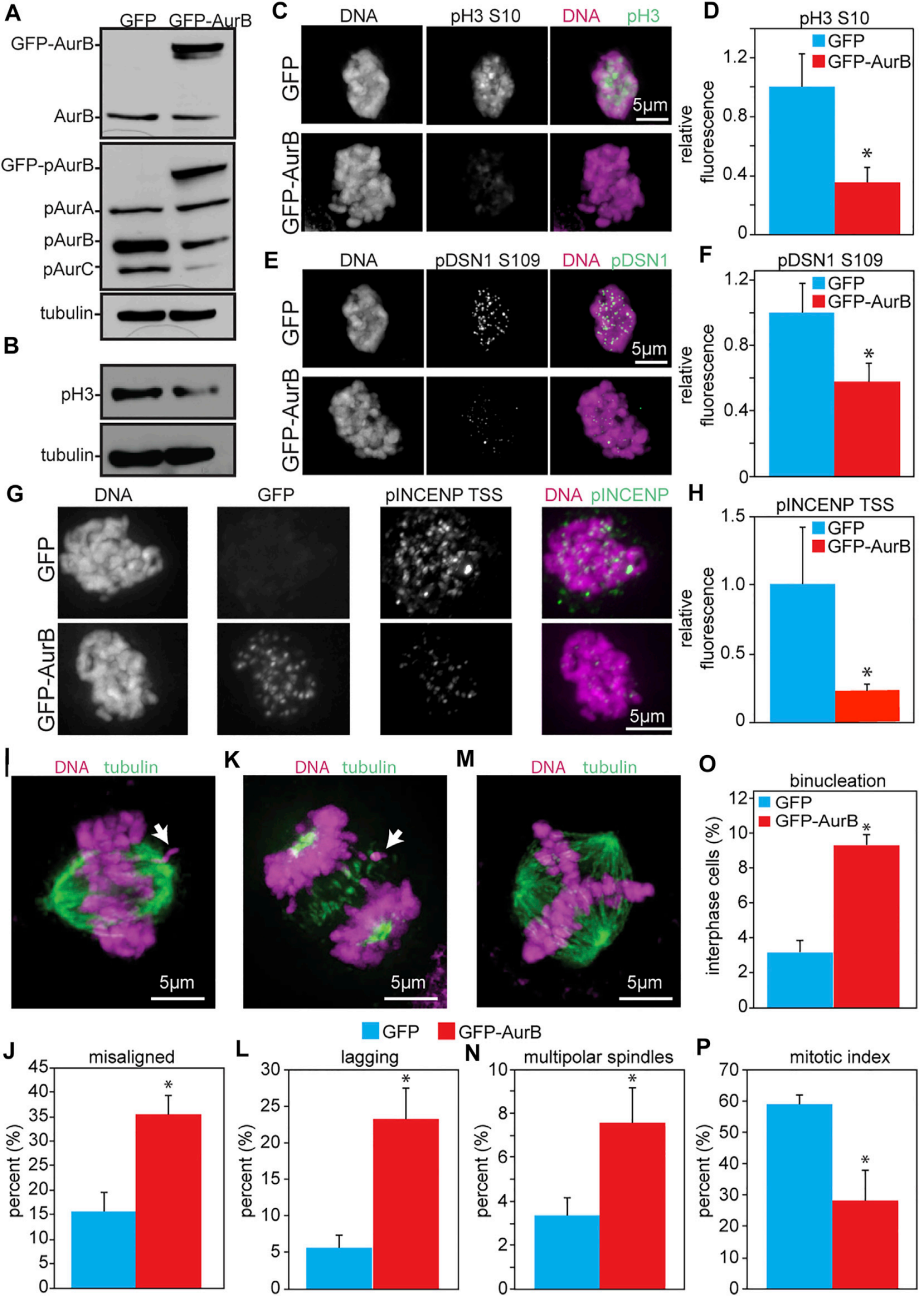
Expression of GFP-Aurora B in HeLa cells causes a reduction in Aurora B kinase activity and an increase in mitotic defects. Cells were analyzed 48 h after infection with retroviruses expressing GFP-Aurora B or GFP alone. **(A)** Immunoblot showing endogenous pT232 (active) Aurora B is reduced in HeLa cells overexpressing Aurora B. Tubulin is shown as a loading control. **(B)** Immunoblot showing reduced phosphorylation of the bona fide Aurora B substrate histone H3 Serine 10 in HeLa cells infected with retroviruses expressing GFP-Aurora. Tubulin is shown as a loading control. **(C–H)** Quantitative immunofluorescence demonstrating that expression of GFP-Aurora B decreases phosphorylation of histone H3 (pH3), the kinetochore component DSN1, and INCENP in HeLa cells. **(C,E,G)** Representative images of pH3, pDSN1, and pINCENP, respectively, in HeLa cells expressing GFP and GFP-Aurora B. **(D,F,H)** Quantification of pH3 S10 **(D)**, pDSN1 S109 **(F)**, and pINCENP TSS **(H)** demonstrating that expression of GFP-Aurora B significantly reduces phosphorylation of these Aurora B substrates in HeLa cells. *n* > 10 cells from each of 3 independent experiments. **(I–P)** GFP-Aurora B expression increases the incidence of mitotic defects. **(I)** Representative image of a misaligned chromosome (arrow). **(J)** Expression of GFP-Aurora B in HeLa cells causes an increase in misaligned chromosomes in metaphase. *n* > 50 metaphase cells from 3 independent experiments. **(K)** Representative image of a lagging chromosome (arrow). **(L)** Expression GFP-Aurora B in HeLa cells causes an increase in lagging chromosomes. *n* > 250 anaphase and telophase cells from each of 3 independent experiments. **(M)** Representative image of a multipolar spindle. **(N)** Expression of GFP-Aurora B in HeLa cells causes an increase in multipolar spindles. *n* > 250 mitotic cells from each of 3 independent experiments. **(O)** Expression of GFP-Aurora B increases the percentage of binucleate HeLa cells. *n* > 250 cells from each of three independent experiments. **(P)** Expression of GFP-Aurora B in HeLa cells impairs their ability to arrest in mitosis after 16 h of treatment with the microtubule poison colcemid, consistent with a weakened mitotic checkpoint. *n* > 250 cells from each of 3 independent experiments. *p < 0.05.

As an initial test of the consequences of increased Aurora B expression in human cells, HeLa cells were infected with retroviruses expressing human Aurora B tagged at the N-terminus with GFP (Figure 3A). This resulted in reduced phosphorylation of pH3 (S10) as detected by blotting and quantitative immunofluorescence (Figures 3B–D). Examination of another phosphorylation site on DSN1 (serine 109) by quantitative immunofluorescence revealed that this site was also less phosphorylated in cells expressing GFP-Aurora B as compared to cells expressing GFP alone (Figures 3E,F). Similarly, phosphorylation of the TSS motif on INCENP was also reduced in cells expressing GFP-Aurora B (Figures 3G,H).

Consistent with a decrease in Aurora B kinase activity, expression of GFP-Aurora B also caused an increase in misaligned chromosomes in cells with a visible metaphase plate (Figures 3I,J), and an increase in chromosomes that lagged behind the segregating masses of DNA in anaphase and telophase (Figures 3K,L). GFP-Aurora B expressing cells also displayed an elevated percentage of binucleate cells, indicative of cytokinesis failure (Figures 3M–O). Because these cells inherit two centrosomes in G1, they would be expected to have an increased incidence of multipolar spindles in the next mitosis, which was indeed observed (Figures 3M,N). Further, when challenged with colcemid for 16 h, HeLa cells expressing GFP-Aurora B showed a significantly reduced mitotic index (Figure 3P), which was not due to reduced number of cells entering mitosis (Supplementary Figure S5A), consistent with a defect in mitotic checkpoint signaling. Thus, these results demonstrate that increased expression of Aurora B causes defects similar to those caused by Aurora B depletion and support a role for Aurora B in mitotic checkpoint signaling independent of its role in error correction.

Kinase dead Aurora B has been shown to act as a dominant negative, resulting in cytokinesis failure (Katayama et al., 1998; Tatsuka et al., 1998; Terada et al., 1998; Ota et al., 2002; Honda et al., 2003; Girdler et al., 2006). To confirm that the exogenously expressed Aurora B in our experiments did indeed have kinase activity, we immunoprecipitated Aurora B-3xFLAG and tested its ability to autophosphorylate and to phosphorylate histone H3 *in vitro* (Supplementary Figure S6). Our Aurora B construct demonstrated INCENP-stimulated kinase activity, as expected for wild type kinase. Together, these data show that increased expression of Aurora B in three different species reduces Aurora B substrate phosphorylation and causes mitotic defects consistent with Aurora B inhibition.

### Increased Aurora B expression in human breast cells causes mitotic defects

Having confirmed that elevated levels of Aurora B protein result in reduced Aurora B substrate phosphorylation and concomitant mitotic defects in HeLa cells, we then tested the consequences of increased Aurora B expression in additional human cell types. MCF10A non-transformed breast cells along with the breast cancer cell lines MDA-MB-231 and Cal51 were infected with retroviruses expressing GFP-tagged Aurora B. Expression of GFP-Aurora B in each of these breast cell lines caused a decreased mitotic index in response to microtubule depolymerization with colcemid, consistent with a weakened mitotic checkpoint (Supplementary Figure S5B). GFP-Aurora B expression also increased the number of binucleate cells in all three cell types, consistent with cytokinesis failure (Supplementary Figure S5C). Reduction of Aurora B kinase activity has previously been shown to cause these defects (Hauf et al., 2003; Santaguida et al., 2011).

**FIGURE 4:**
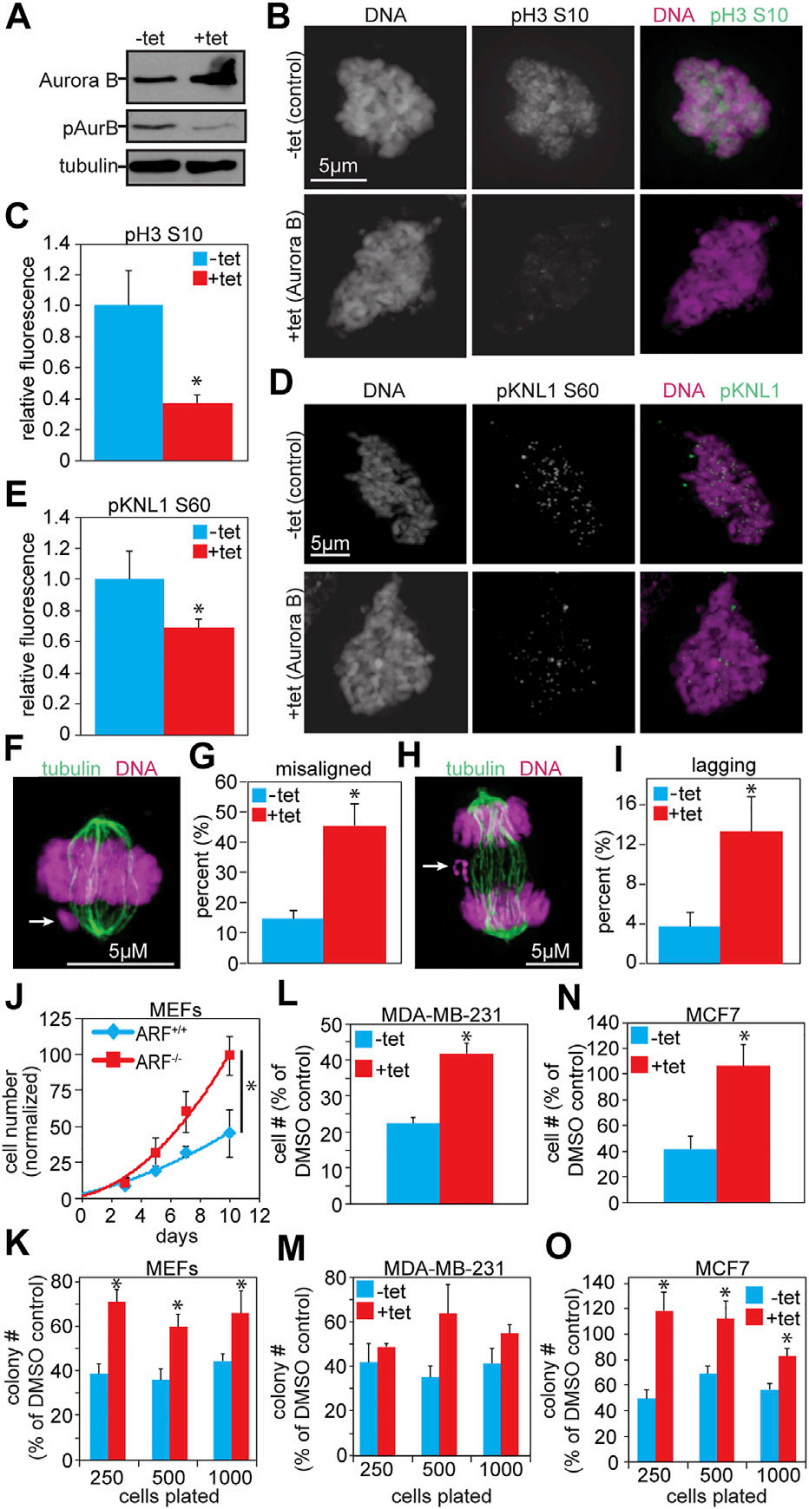
Increased expression of untagged Aurora B inhibits Aurora B kinase activity and causes resistance to Aurora kinase inhibition. **(A)** Immunoblot showing ≥2- fold overexpression of untagged Aurora B in MDA-MB-231 cells treated with tetracycline. Despite increased protein levels of Aurora B, pT232 (active) Aurora B is reduced. Tubulin is shown as a loading control. **(B–E)** Quantitative immunofluorescence showing that increased Aurora B expression results in reduced phosphorylation of the Aurora B substrates histone H3 and KNL1. **(B,D)** Representative images of pH3 and pKNL1 in MDA-MB-231 cells ± 48 h of tet-inducible expression of Aurora B **(C,E)** Quantification of B and D. *n* > 10 cells from each of 3 independent experiments. **(F–I)** 48 h of tet-induced Aurora B expression induces mitotic defects in MDA-MB-231 cells. **(F)** Representative image of a misaligned chromosome (arrow). **(G)** Elevated Aurora B expression increases the incidence of misaligned chromosomes. *n* > 50 metaphases from 3 independent experiments. **(H)** Representative image of a lagging chromosome (arrow). **(I)** Increased Aurora B expression induces lagging chromosomes. *n* > 100 anaphase and telophases from 3 independent experiments. **(J–O)** Increased Aurora B expression causes resistance to Aurora B inhibition. **(J)** Primary ARF^−/−^ MEFs grow significantly better than ARF^+/+^ MEFS after 16 h of exposure to the Aurora B inhibitor ZM447439. Cell number shown is normalized to the number of ARF^+/+^ cells 10 days after treatment with DMSO. *n* = 3 independent experiments. **(K)** Primary ARF^−/−^ MEFs (red), which overexpress endogenous Aurora B, have significantly improved colony forming ability as compared to ARF^+/+^ MEFs (blue) after 16 h exposure to ZM447439. *n* = 3 independent experiments. **(L)** MDA-MB-231 cells expressing untagged Aurora B in response to tetracycline grow significantly better over the course of 10 days than controls after 16 h exposure to ZM447439. *n* = 3 independent experiments. **(M)** MDA-MB-231 cells expressing tet-inducible wild type Aurora B exhibit significantly elevated colony forming ability compared to control MDA-MB-231 cells after exposure to ZM447439. *n* = 3 independent experiments. **(N)** MCF7 cells expressing Aurora B–GFP in response to tetracycline grow significantly better over the course of 10 days than MCF7 controls after treatment with ZM447439. *n* = 3 independent experiments. **(O)** MCF7 cells expressing Aurora B–GFP form significantly more colonies than control cells after exposure to ZM447439. *n* = 3 independent experiments. *p < 0.05.

To determine whether the addition of an N- or C-terminal tag onto Aurora B was responsible for the apparent inhibition of Aurora B kinase activity, we generated MDA-MB-231 cells that stably express untagged Aurora B in response to tetracycline. 48 h of tetracycline treatment resulted in elevated levels of Aurora B protein, but reduced autophosphorylation at T232, indicating reduced Aurora B activity (Figure 4A). Consistent with this, phosphorylation of histone 3 (S10) was also reduced, as assessed by quantitative immunofluorescence (Figures 4B,C). Furthermore, an additional Aurora B substrate and KMN-complex member, KNL1, had reduced phosphorylation at serine 60 (Figures 4D,E). Thus, a modest increase in expression of untagged Aurora B in human breast cancer cells reduced Aurora B substrate phosphorylation.

Increased expression of untagged Aurora B in MDA-MB-231 cells caused a range of mitotic defects consistent with inhibition of Aurora B activity. These included significant increases in misaligned (Figures 4F,G) and lagging chromosomes (Figures 4H,I). Mitotic cells expressing elevated levels of Aurora B also displayed an increase in multipolar spindles (Supplementary Figures S5D,E), suggesting a prior cytokinesis failure. Consistent with this, the population of binucleate cells was significantly higher in cells expressing elevated Aurora B than in control cells (Supplementary Figures S5F,G). Additionally, cells expressing increased Aurora B were impaired in their ability to arrest in mitosis in response to microtubule disruption, consistent with weakened mitotic checkpoint signaling (Supplementary Figures S5H). Thus, exogenous expression of N-terminally tagged, C-terminally tagged, and untagged Aurora B each reduced Aurora B substrate phosphorylation and induced mitotic defects associated with Aurora B kinase inhibition.

### Phenotypes caused by increased expression of Aurora B are not due to ectopic phosphorylation of cytoplasmic substrates

Increased expression of Aurora B reduced autophosphorylation of Aurora B as well as phosphorylation of substrates at the inner centromere (INCENP TSS), kinetochore (DSN S100, S109, KNL S60) and chromosome (pH3 S10). The resulting phenotypes are consistent with those caused by inhibition or depletion of Aurora B (Ditchfield et al., 2003; Hauf et al., 2003). However, it remained possible that increased expression of Aurora B led to increased cytoplasmic activity of Aurora B, which was responsible for the mitotic and cytokinetic defects observed. Aurora B phosphorylation of vimentin S72 (Goto et al., 2003) and desmin S60 (Kawajiri et al., 2003) is normally restricted to the midbody during telophase. As a test for the cytoplasmic Aurora B kinase activity in cells expressing elevated levels of Aurora B, phosphorylation of vimentin S72 and desmin S60 was assayed in MDA-MB-231 cells expressing tet-inducible untagged Aurora B. Phosphorylation of vimentin and desmin was not elevated by induced Aurora B expression (Supplementary Figures S7A–D), supporting the conclusion that defects caused by increased Aurora B expression are due to reduced phosphorylation of Aurora B substrates, rather than ectopic phosphorylation of inappropriate cytoplasmic substrates.

### Increased Aurora B expression causes resistance to Aurora B inhibition in mouse and human cells

Tumors overexpressing Aurora B are expected to have increased Aurora B substrate phosphorylation and to be addicted to the enhanced oncogenic signal from elevated Aurora B kinase activity, making them hypersensitive to Aurora kinase inhibitors, several of which have entered clinical trials (Altieri, 2008; Boss et al., 2009; Kantarjian et al., 2013a; Kantarjian et al., 2013b). However, primary ARF^−/−^ MEFs, which express elevated levels of endogenous Aurora B, actually showed resistance to the Aurora B kinase inhibitor ZM447439 in population growth assays (Figure 4J). ARF^−/−^ MEFs also exhibited enhanced colony forming ability relative to ARF^+/+^ MEFs after treatment with ZM447439 (Figure 4K). We note that ARF loss causes a proliferative advantage (Supplementary Figures S8A,B; (Fontana et al., 2019)), but the effect on resistance to ZM447439 persists even after accounting for this (Figures 4J,K). Expression of tagged or untagged Aurora B did not have a substantial impact on proliferation at the population or single cell level (Supplementary Figures S8C–F). Interestingly, similar to ARF^−/−^ MEFs with increased expression of Aurora B, MDA-MB-231 cells stably expressing untagged Aurora B in response to tetracycline, as well as MCF7 breast cancer cells stably expressing Aurora B tagged with the mNeonGreen version of GFP at the C-terminus in response to tetracycline, also exhibited improved growth in response to ZM447439 at both the population and single cell levels (Figures 4L–O). Thus, cells expressing increased levels of Aurora B are less, rather than more, sensitive to Aurora B kinase inhibition.

### Co-expression of INCENP with Aurora B restores Aurora B kinase activity and rescues mitotic defects

**FIGURE 5:**
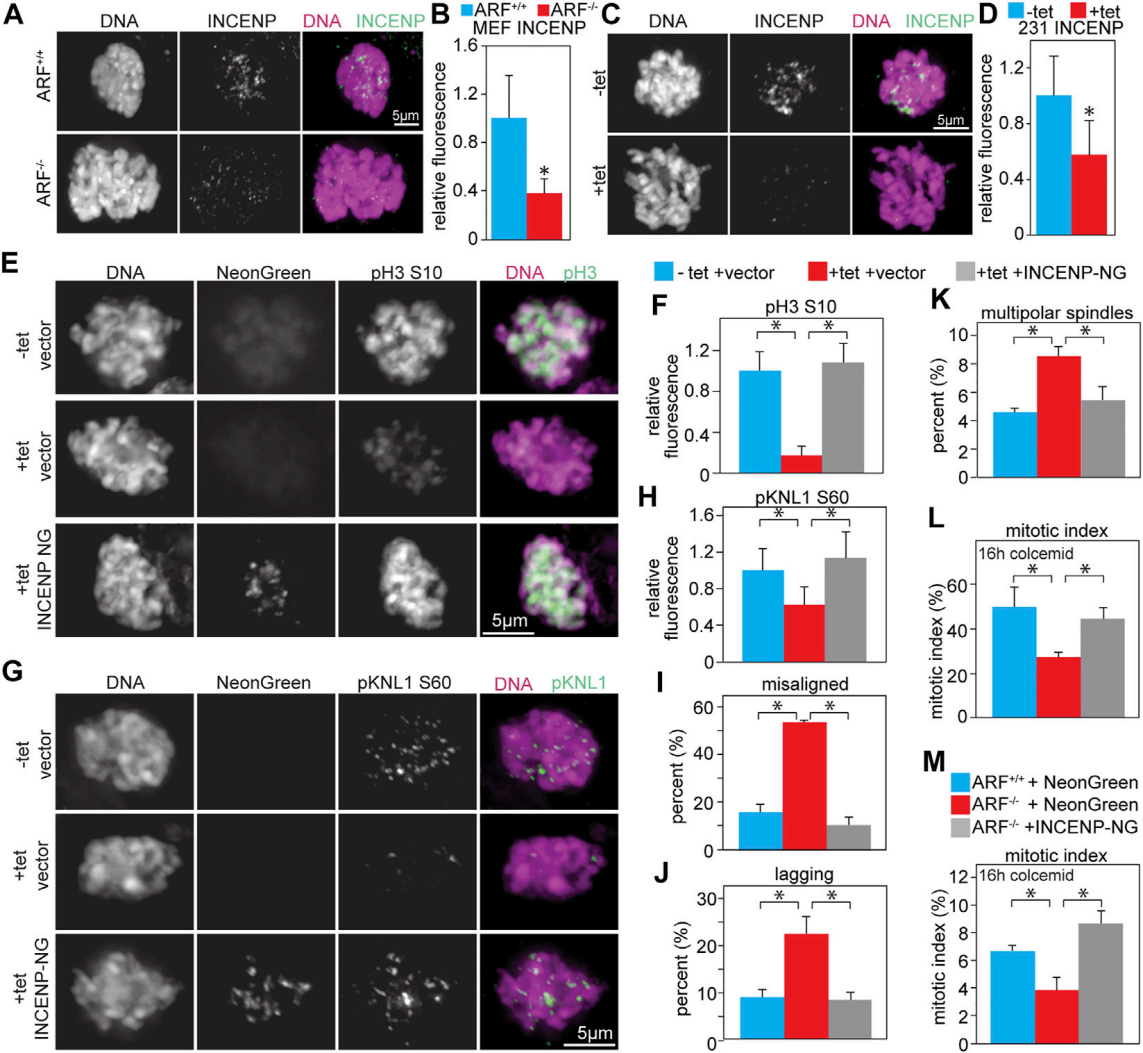
Co-expression of INCENP restores Aurora B kinase activity and rescues mitotic defects. **(A–D)** Upregulation of Aurora B mislocalizes INCENP. **(A)** ARF^−/−^ MEFs, which have increased levels of endogenous Aurora B, have reduced INCENP localization to the inner centromere. **(B)** Quantification of INCENP levels in primary MEFs. *n* > 10 cells from each of 3 independent experiments. **(C)** Tet-inducible Aurora B expression in MDA-MB-231 cells reduces INCENP localization to the inner centromere. **(D)** Quantification of INCENP levels in MDA-MB-231 cells. *n* > 10 cells from each of 3 independent experiments. **(E–L)** Infection with lentiviruses expressing INCENP-mNeonGreen (48 h) rescues Aurora B inhibition and mitotic defects caused by tet-induced upregulation of Aurora B (48 h). **(E–H)** Aurora B upregulation reduces phosphorylation of Aurora B substrates, but co-expression of INCENP-mNeonGreen restores phosphorylation of pH3 **(E–F)** and pKNL1 **(G–H)**. **(F,H)** Quantification of pH3 **(F)** and pKNL **(H)** levels. *n* > 10 cells from each of 3 independent experiments. **(I–J)** Upregulation of Aurora B increases the incidence of misaligned **(I)** and lagging **(J)** chromosomes, while expression of INCENP-mNeonGreen in conjunction with Aurora B upregulation rescues this defect. n > 50 metaphase cells **(I)** or 100 anaphase and telophase cells **(J)** from 3 independent experiments. **(K)** Aurora B overexpression increases the incidence of multipolar spindles, but co-expression of INCENP rescues this defect. *n* > 250 mitotic cells from each of 3 independent experiments. **(L)** Upregulation of Aurora B impairs mitotic checkpoint signaling, resulting in a reduced mitotic index after 16 h of colcemid treatment. Expression of INCENP-mNeonGreen in conjunction with Aurora B upregulation rescues this defect. *n* > 250 cells from each of 3 independent experiments. **(M)** ARF^−/−^ MEFs, which have elevated protein levels of endogenous Aurora B, have an impaired ability to arrest in mitosis after 16 h of treatment with the microtubule poison colcemid. However, expression of INCENP-mNeonGreen restores mitotic checkpoint signaling in ARF^−/−^ MEFs. *n* > 250 cells from each of 3 independent experiments. *p < 0.05.

INCENP stimulates Aurora B kinase activity ∼10 fold *in vitro* (Honda et al., 2003). Previous studies from the Inagaki lab (Kawajiri et al., 2003; Yasui et al., 2004) found that co-expression of Myc-Aurora B with HA-INCENP caused abnormal phosphorylation of histone H3 in ∼90% of interphase cells, while expression of Myc-Aurora B alone had no effect, suggesting that co-expression of INCENP could increase Aurora B substrate phosphorylation. Consistent with this idea, complexes of Aurora B and INCENP autophosphorylate in trans (Sessa et al., 2005; Rosasco-Nitcher et al., 2008; Elkins et al., 2012; Zaytsev et al., 2016). Increasing the local concentration of Aurora B:INCENP complexes enhances Aurora B kinase activity in *Xenopus* extracts and human cells (Kelly et al., 2007; Wang et al., 2011), suggesting that decreasing the local concentration of Aurora B:INCENP complexes would reduce Aurora B kinase activity. Both ARF^−/−^ MEFs, which have elevated levels of endogenous Aurora B, and MDA-MB-231 cells expressing elevated levels of untagged Aurora B in response to tetracycline had significantly reduced levels of INCENP at inner centromeres/kinetochores (Figures 5A–D), but equivalent levels of total INCENP protein compared to relevant controls (Supplementary Figures S7E–H). This mislocalization is consistent with a decrease in the local concentration of Aurora B:INCENP complexes at inner centromeres/kinetochores, which would be expected to result in lower activity of a kinase that autophosphorylates in trans.

To determine if co-expression of INCENP with Aurora B could rescue Aurora B kinase activity, MDA-MB-231 cells stably expressing untagged Aurora B in response to tetracycline were infected with lentiviruses expressing INCENP-mNeonGreen. While increased expression of Aurora B in isolation reduced phosphorylation of histone H3 (S10) and KNL1 (S60), co-expression of INCENP restored the phosphorylation of these substrates (Figures 5E–H).

We next sought to determine whether mitotic defects observed in Aurora B overexpressing cells were rescued upon co-expression of INCENP. Both misaligned and lagging chromosomes were increased in cells expressing elevated Aurora B alone, but co-expression of INCENP reduced these to control levels (Figures 5I,J). Elevated expression of Aurora B increased the incidence of multipolar spindles in MDA-MB-231 cells, while co-expression of INCENP reduced these (Figure 5K). Additionally, the reduced mitotic index in response to microtubule depolymerization caused by increased Aurora B expression was rescued by co-expression of INCENP (Figures 5L,M). Together, these data demonstrate that an increased level of INCENP is necessary to prevent increased expression of Aurora B from reducing phosphorylation of Aurora B substrates.

### Expression of INCENP is sufficient to increase Aurora B activity in cells endogenously expressing elevated levels of Aurora B

**FIGURE 6:**
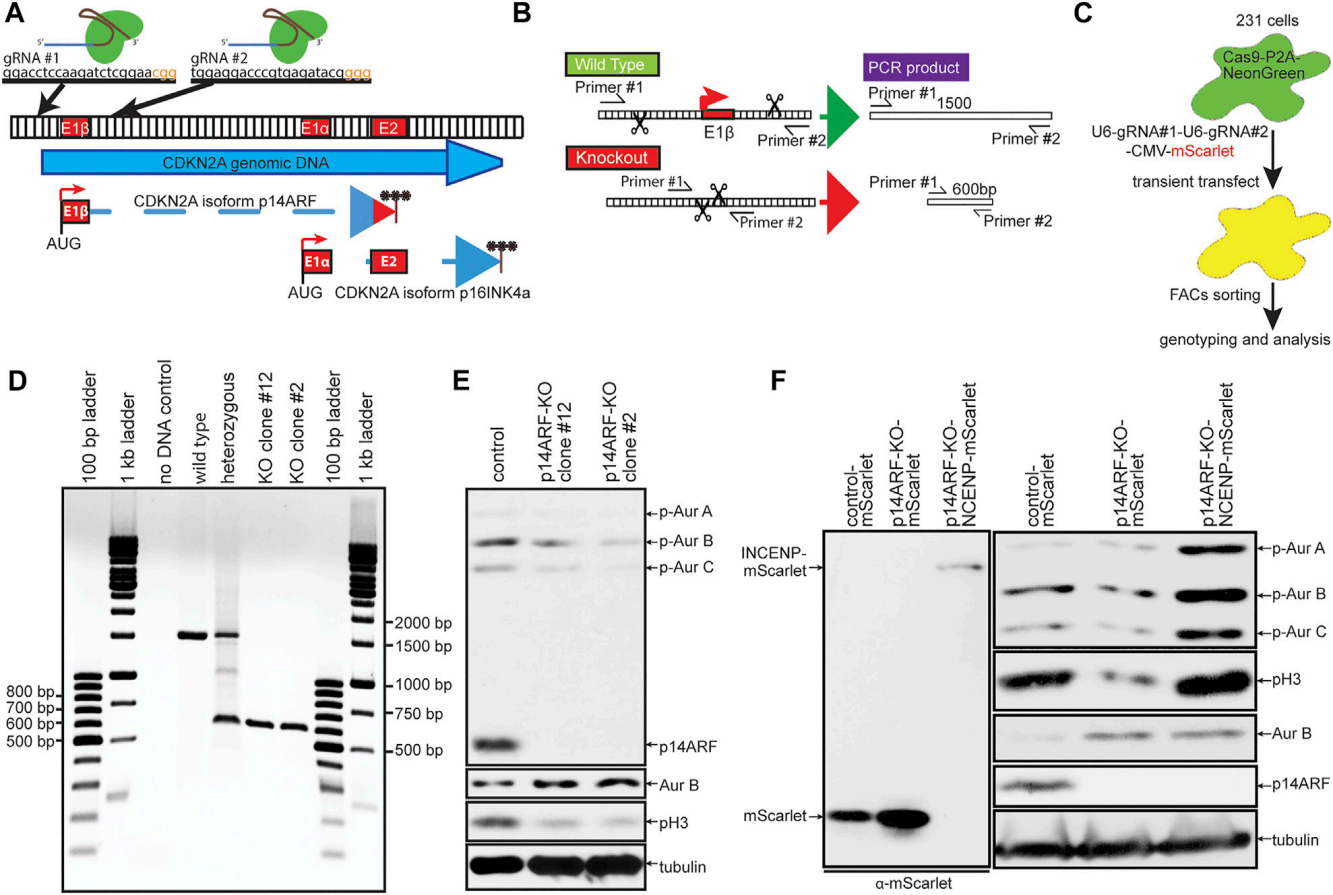
Expression of INCENP is sufficient to increase Aurora B activity in cells endogenously expressing elevated levels of Aurora B. **(A–C)** Schematic diagram of CRISPR-Cas9 p14ARF knockout (KO) strategy. **(A)** The CDKN2A genomic locus encodes for p14ARF and p16INKA. Two guide RNAs targeting exon1β of CDKN2A were used to produce specific p14ARF KO. **(B)** PCR strategy for confirming successful KO of p14ARF. **(C)** MDA-MB-231 cells expressing Cas9-P2A-NeonGreen were transiently transfected with a construct expressing mScarlet and multiple guide RNAs and FACs sorted for mScarlet-NeonGreen double positive cells before genomic analysis for p14ARF knockout. **(D)** Double positive cells were screened using the PCR strategy in **(B)**. Homozygous KO clones show a 600 bp DNA band. **(E)** p14ARF KO clones 2 and 12 show complete KO of p14ARF by immunoblot, which induces constitutively elevated levels of endogenous Aurora B and lower levels of pH3. **(F)** p14ARF KO increases Aurora B and reduces phosphorylation of Aurora B and pH3. Expression of INCENP-mScarlet restores endogenous Aurora B kinase activity.

Co-expression of INCENP was sufficient to restore Aurora B substrate phosphorylation and rescue mitotic defects in MDA-MB-231 cells induced to express elevated levels of Aurora B. However, it remained unclear whether phosphorylation of Aurora B substrates in cells with endogenously elevated expression of Aurora B could also be increased by expression of INCENP. To test this, we used CRISPR/Cas9 editing to generate ARF^−/−^ MDA-MB-231 cells (Figures 6A–E). As in primary MEFs, ARF knockout increased expression of Aurora B but decreased active, phosphorylated Aurora B and pH3 (Figures 6E,F). Expression of INCENP-mScarlet in ARF^−/−^ cells restored phosphorylation of pH3 and Aurora B to levels in ARF^+/+^ cells (Figure 6F). Thus, increasing INCENP expression can restore Aurora B activity in cells that endogenously express heightened levels of Aurora B.

### Aurora B expression in human breast cancers correlates with Survivin and Borealin but not with INCENP

**FIGURE 7:**
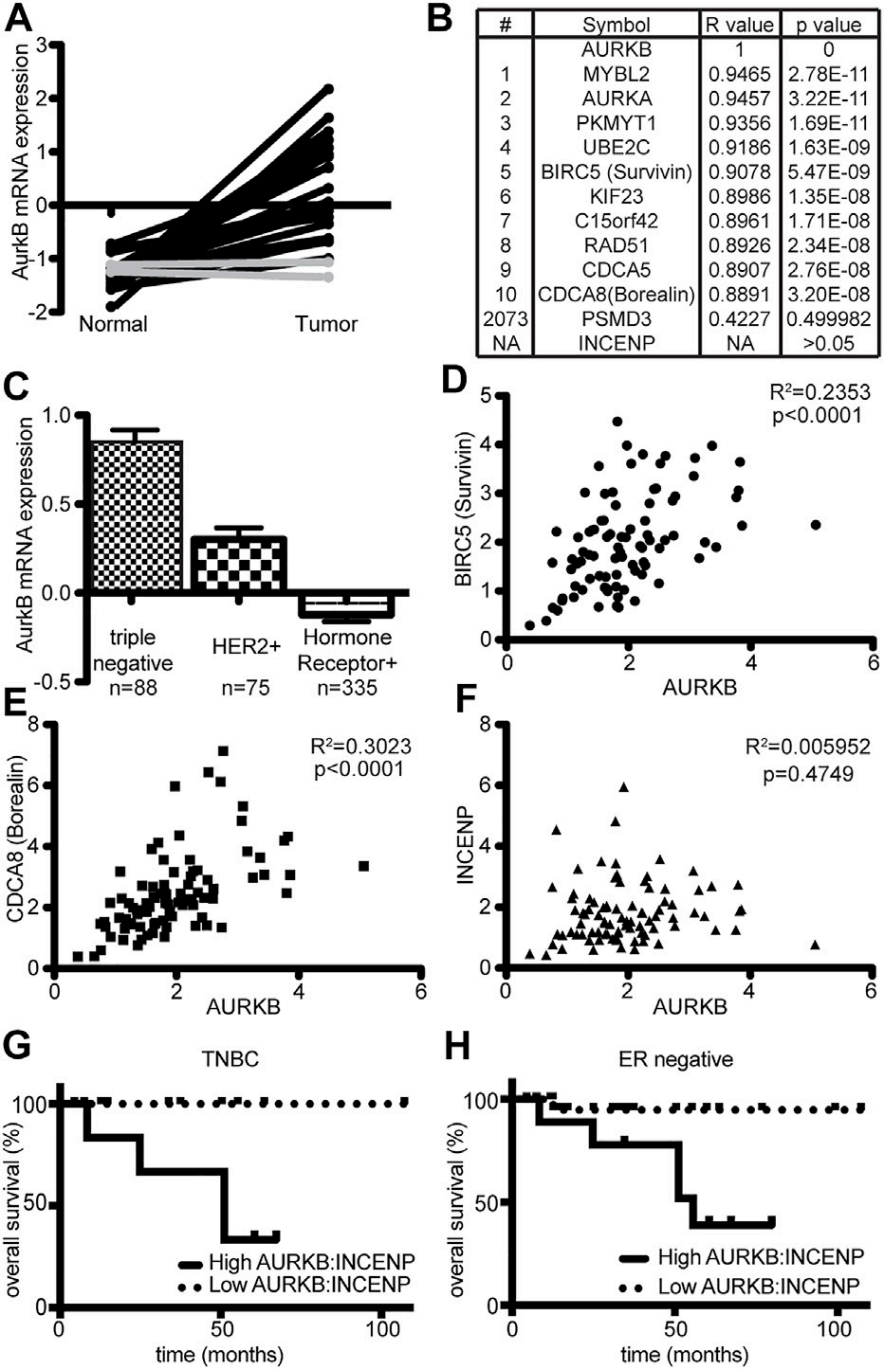
Aurora B expression correlates with Survivin and Borealin, but not INCENP, in breast cancer. Analysis of TCGA breast cancer data (Network, 2012). **(A)** Analysis of AURKB (Aurora B) mRNA expression levels in 22 matched pairs of normal and cancerous breast tissue. Lines connect dots displaying the AURKB expression level in matching tissue. In 20 of 22 cases, expression of AURKB mRNA is substantially higher in tumor tissue than in matched normal breast tissue (black). The other two cases are shown in gray. **(B)** Pavlidis Template Matching was used to identify genes whose expression most strongly correlates with AURKB in the 22 tumor samples in **(A)**. Expression of BIRC5 (Survivin) and CDCA8 (Borealin) is highly correlated with that of AURKB. INCENP is absent from the 468 genes whose expression correlated with AURKB to a corrected *p* value < 0.05. The full list of genes is shown in Supplementary Table S1. **(C)** Stratification of all breast cancers classified with respect to subtype showing that AURKB is most highly expressed in triple negative breast cancers. **(D–F)** Dot plots of mRNA expression values of AURKB with BIRC5 **(D)**, CDCA8 **(E)**, and INCENP **(F)** showing that AURKB expression correlates with BIRC5 and CDCA8 but not with INCENP in triple negative breast cancers. Pavlidis Template Matching further demonstrates that BIRC5 and CDCA8 expression correlate with AURKB, while INCENP expression does not (see Supplementary Table S2). **(G)** Kaplan-Meier curves for overall patient survival of high or low AURKB:INCENP ratio expressing tumors. Triple Negative Breast Cancers (TNBC) were divided into those exhibiting an AURKB:INCENP protein ratio of >1.2 or less than 1.2. TNBC tumors with an AURKB:INCENP ratio >1.2 have significantly more aggressive disease that those with a ratio <1.2 (Log Rank test *p* = 0.0376). **(H)** Same as in **(G)** but using histologically ER-tumors. ER-tumors with a high AURKB:INCENP ratio show an aggressive disease progression (*p* = 0.0253).

Although Aurora B is frequently overexpressed in human tumors, cancers often misregulate multiple genes in concert. Therefore, tumors that overexpress Aurora B may also co-overexpress INCENP. To determine whether INCENP expression was increased in parallel with Aurora B expression, we analyzed breast cancer data from The Cancer Genome Atlas (Network, 2012). Matched pairs of normal and cancerous breast tissues were examined for Aurora B (AURKB) transcript levels (Figure 7A). In 20 of 22 cases, breast tumors showed markedly increased expression of AURKB compared to normal tissues. We next sought to correlate Aurora B expression levels with other genes using Pavlidis Template Matching (Pavlidis and Noble, 2001). The expression of 468 genes was found to correlate with AURKB in these 22 tumors to a corrected *p* value of <0.05 (Supplementary Table S1). The chromosomal passenger complex (CPC) components Survivin (BIRC5) and Borealin (CDCA8), which form a complex with Aurora B and INCENP, were included in the top 10 genes whose expression correlated with AURKB (Figure 7B). However, INCENP did not appear on the list of genes whose expression correlated with AURKB.

AURKB is most highly expressed in the triple negative subset of breast cancers (Figure 7C). This subtype lacks expression of estrogen and progesterone receptors, does not overexpress HER2 receptors, and is an aggressive tumor subtype with limited treatment options and poor prognosis. Pavlidis Template Matching was used to identify the genes whose expression correlated with AURKB in triple negative breast cancer. Again, expression of the CPC genes BIRC5 and CDCA8 highly correlated with AURKB expression while INCENP expression did not (Supplementary Table S2). Pairwise comparison of the expression of AURKB with BIRC5, CDCA8 and INCENP again revealed a close correlation between the expression of AURKB with BIRC5 and CDCA8 in individual tumors (Figures 7D,E), while no such correlation existed between AURKB and INCENP (Figure 7F). This indicates that most breast tumors overexpressing Aurora B do not co-overexpress INCENP, strongly suggesting that these cancers have reduced, rather than elevated, Aurora B kinase activity. Importantly, patients whose tumors had high ratios of Aurora B:INCENP protein expression, consistent with overexpression of Aurora B without concomitant overexpression of INCENP, showed substantially poorer survival outcomes than patients in which the ratio of Aurora B:INCENP expression was lower (Figures 7G,H). These data demonstrate that Aurora B overexpression without concomitant INCENP overexpression is common in breast tumors, where it correlates with poor prognosis.

## Discussion

Increased expression of Aurora B at the mRNA and protein levels has been recognized as a common characteristic of human cancers for two decades. Elevated protein levels of Aurora B are anticipated to result in increased kinase activity, and a key rationale for the clinical development of Aurora B inhibitors was that elevated kinase expression suggests dependence of cancer on Aurora B kinase activity. However, clinical experience with Aurora B inhibitors has been largely disappointing (Boss et al., 2009). We show here that, contrary to expectations, increased expression of Aurora B inhibits rather than increases its kinase activity against six substrates (Aurora B T232, histone H3 S10, DSN1 S100, DSN1 S109, INCENP TSS, KNL1 S60) across three species (human, mouse, and worm). Cells expressing elevated Aurora B protein levels display mitotic defects previously shown to occur due to Aurora B inhibition or depletion (Ditchfield et al., 2003; Hauf et al., 2003; Carmena et al., 2012), including misaligned and lagging chromosomes, weakened mitotic checkpoint signaling, and cytokinesis failure. These data show that increased expression of species-appropriate Aurora B causes the same phenotypes as decreased expression of Aurora B.

The decrease in Aurora B kinase activity caused by increased expression of Aurora B can be rescued by co-expression of INCENP. However, since most breast cancers that overexpress Aurora B do not show similarly elevated levels of INCENP, these cancers are likely to have reduced, rather than elevated, levels of Aurora B kinase activity. Moreover, increased expression of Aurora B mediates resistance, rather than hypersensitivity, to Aurora B inhibitors. This suggests that, while Aurora B expression alone is insufficient to predict response to Aurora B kinase inhibitors, the ratio of Aurora B and INCENP expression could be informative.

Aurora B plays a central role in error correction, releasing inappropriate connections between kinetochores and microtubules that do not result in tension (Vader et al., 2008). Whether Aurora B activates mitotic checkpoint signaling solely by releasing inappropriate attachments to produce unattached kinetochores during error correction or participates directly in checkpoint signaling has been debated in the field. However, on balance, recent evidence favors the conclusion that Aurora B functions in mitotic checkpoint signaling independent of error correction in multiple species (Kallio et al., 2002; Petersen and Hagan, 2003; Vader et al., 2007; Santaguida et al., 2011; Hadders et al., 2020; Roy et al., 2022). Our evidence supports this conclusion, and demonstrates that increased Aurora B, which reduces Aurora B substrate phosphorylation, also weakens mitotic checkpoint signaling in response to unattached kinetochores.

Previous studies came to contradictory conclusions about whether increased levels of Aurora B induce mitotic or cytokinetic defects (Katayama et al., 1998; Terada et al., 1998; Murata-Hori et al., 2002; Ota et al., 2002; Honda et al., 2003; Girdler et al., 2006). These studies were predominantly focused on consequences of kinase dead versions of Aurora B, with wild type Aurora B included as a control that uniformly showed subtler phenotypes than kinase dead Aurora B. Here, a focus on wild type Aurora B has revealed that increased expression does indeed cause defects in chromosome congression and segregation, mitotic checkpoint signaling, and cytokinesis. Moreover, we report the novel finding that these defects are due to a quite unexpected decrease in Aurora B substrate phosphorylation in cells with elevated levels of Aurora B protein.

Consistent with standard expectations, but in contrast to our findings here, exogenous expression of N-terminally tagged Aurora B was previously reported to increase phosphorylation of serine 10 on histone H3 (Ota et al., 2002; Girdler et al., 2006). The reason(s) for the difference in this phenotype remain unclear. In the previously published work, FLAG-tagged AIM1/Aurora B was expressed in Chinese hampster embryo fibroblasts (Ota et al., 2002). Based on the publication cited in the Ota et al. manuscript (Tatsuka et al., 1998), it appears human AIM1 with an alanine to serine mutation at residue 294 was expressed. It is unclear to what extent this mutation, which is C-terminal to the kinase domain and outside of the INCENP binding domain, affects protein function.

Mice in which the promoter of one allele of endogenous Aurora B has been replaced with a tetracycline inducible promoter (AurB^+/tet^) have been generated (González-Loyola et al., 2015). Importantly, in the absence of tetracycline the AurB^+/tet^ cells are essentially heterozygous for Aurora B since the endogenous promoter was replaced with the tetracycline-inducible one. Consistent with this, in the absence of tetracycline, AurB^+/tet^ cells have lower levels of Aurora B protein than AurB^+/+^ cells (González-Loyola et al., 2015). Since cells expressing lower levels of Aurora B, either due to genetic knockout (Fernández-Miranda et al., 2011) or siRNA (Hauf et al., 2003), were previously shown to have reduced Aurora B kinase activity, we anticipate that AurB^+/tet^ cells in the absence of tetracycline—which were used as controls—have lower levels of Aurora B substrate phosphorylation than wild type cells. Although a substantial increase in Aurora B substrate phosphorylation was expected after doxycycline induced Aurora B overexpression, phosphorylation of pH3 and the kinetochore substrate Hec1 (serine 55) was similar in AurB^+/tet^ cells with and without tetracycline. We hypothesize this is because AurB^+/tet^ cells in the absence of tetracycline, which have lower levels of Aurora B expression, and AurB^+/tet^ cells in the presence of tetracycline, which have higher levels of Aurora B, both have lower levels of Aurora B activity than AurB^+/+^ cells.

Overall, our data suggest a model in which the cellular ratio of Aurora B to INCENP is critically important for full Aurora B activity. Increased expression of Aurora B titrates INCENP away from its proper localization at the inner centromere/kinetochore, decreasing the local concentration of Aurora B-INCENP complexes. Aurora B-INCENP complexes have previously been shown to autophosphorylate in trans (Sessa et al., 2005; Rosasco-Nitcher et al., 2008; Elkins et al., 2012), and locally increasing the concentration of these complexes enhances Aurora B kinase activity (Kelly et al., 2007; Wang et al., 2011). Endogenous levels of Aurora B and INCENP permit efficient activation of these complexes in trans, resulting in a substantial increase in Aurora B kinase activity during mitosis (Supplementary Figure S9A). Increased expression of Aurora B results in mislocalization of INCENP and dilutes the local concentration of Aurora B-INCENP complexes at inner centromeres/kinetochores, resulting in a net decrease in Aurora B autophosphorylation at T232 and the phosphorylation of Aurora B downstream targets (Supplementary Figure S9B). Aurora B binds to the C terminus of INCENP, while the N terminus of INCENP binds to Survivin and Borealin (Ruchaud et al., 2007) to form the CPC. Localization of the CPC to the centromeric region is mediated by Survivin, which binds to threonine 3 in histone H3 after it is phosphorylated by Haspin (Kelly et al., 2010), and Borealin, which binds Shugoshin recruited to histone H2A after phosphorylation on threonine 120 by Bub1 (Yamagishi et al., 2010). Since Aurora B-INCENP complexes localize to the centromeric region through their interaction with Survivin and Borealin, it is unclear how monomeric Aurora B displaces Aurora B incorporated into the CPC from the centromeric region. This will be an interesting topic for future study. Together, these data demonstrate that increased expression of Aurora B inhibits, rather than increases, its associated kinase activity and suggest that the ratio of Aurora B to INCENP expression may be useful in selecting patients likely to respond to Aurora B kinase inhibitors that have entered clinical trials.

## Materials and methods

### Animals, cell culture, and treatments

Animals were maintained in a C57BL/6 background and handled in accordance with the policies of the Institutional Animal Care and Use Committee of the University of Wisconsin–Madison. Primary MEFs were generated from embryonic day 14.5 (E14.5) embryos in 6-cm dishes with 3 ml of chilled 0.05% trypsin/EDTA. After the head, liver, and tail (for genotyping) were removed, embryos were minced using scissors, pipetted up and down using a 10-ml pipette, and incubated at 37°C for 15 min. Embryos were pipetted up and down with an additional 2 ml of trypsin/EDTA and a 5-ml pipette before further incubation at 37°C for 10 min. The solution was transferred to a 15-ml conical tube with 5 ml of primary MEF media. After permitting debris to settle for 1–2 min, supernatant was transferred to a second 15 ml conical tube. MEFs in the supernatant were pelleted and resuspended in high-glucose DMEM (Invitrogen, Carlsbad, CA, United States) containing 15% fetal calf serum (FCS; Sigma-Aldrich, St. Louis, MO, United States), 0.1 mM nonessential amino acids (Invitrogen), 1 mM sodium pyruvate (Invitrogen), 1 μM 2-mercaptoethanol (Acros Biological/Thermo Fisher, Waltham, MA), 2 mM L-glutamine (Invitrogen), and 50 μg/ml penicillin/streptomycin (Invitrogen) and cultured in 3% O_2_ and 10% CO_2_ at 37°C. Experiments were performed on MEFs between passages 3 and 20. HeLa, 293T, and Cal51 cells were grown in high-glucose DMEM, 10% fetal bovine serum (FBS; Sigma-Aldrich, St. Louis, MO), 2 mM L-glutamine, and 50 μg/ml penicillin/streptomycin and cultured in 5% CO_2_ at 37°C. MDA-MB-231 cells were grown in high-glucose DMEM, 10% fetal bovine serum (FBS; Sigma-Aldrich, St. Louis, MO, United States), 2 mM L-glutamine, and 50 μg/ml penicillin/streptomycin and cultured in 10% CO_2_ at 37°C. MCF10A cells were grown in DMEM/F12 with 5% horse serum (Gibco), 20 ng/ml EGF, 1 mg/ml hydrocortisone, 1 mg/ml cholera toxin, 10 mg/ml insulin, and 50 μg/ml penicillin/streptomycin and cultured in 5% CO_2_ at 37°C.

Unless otherwise indicated, colcemid (Enzo, Farmingdale, NY, United States) was used at 100 ng/ml, and mitotic indices were collected after 16 h of treatment. To induce expression of the Aurora B transgene, MDA-MB-231 TetR Aurora B cells were treated with 1 μM tetracycline for 48 h before analysis. Retroviruses were added along with 8 μg/ml Polybrene 48 h before harvest of cells. Transfections were performed using FuGene 6 (Promega).

For colony formation assays, the indicated number of cells were plated in 6-well plates. After 2 days, cells were treated with DMSO or 2 µM ZM447439 (Sigma-Aldrich; 4 µM for MEFs) for 16 h, washed, and allowed to grow for 2 weeks before methanol fixation and counting of crystal violet stained colonies. 16 h of drug treatment was used to simulate the transient drug exposure of patient tumors. For growth assays, 50,000 MEFs were plated and treated the following day with DMSO or 4 µM ZM447439 (Sigma-Aldrich) for 16 h. Cell number was counted using a hemacytometer.

Transgenic *C. elegans* overexpressing GFP-AIR-2 were generated using a biolistic particle delivery system, followed by manual selection using confocal microscopy to identify animals that exhibited the most intense GFP fluorescence in the germline.

### Kinase assays

Kinase activity of HsAurora B-3xFLAG was determined after infection in HEK293T cells. Cells singly infected with 3xFLAG were included as a negative control. Aurora B-3xFLAG was immunoprecipitated with M2-agarose slurry (Selleck B23102) and incubated in buffer (200 mM Tris-HCl, pH 7.5, 100 mM MgCl_2_, 1.5M NaCl, 100 mM NaF) with 1 mM DTT, 1 μM cold ATP, 5 μCi [γ-^32^P] ATP, 2.5 μg histone H3 (NEB M2506S) and with or without the IN-box of recombinant INCENP (Signal Chem I30-31H) at 30°C for 30 min and resolved by SDS-PAGE. γ-^32^P incorporation was visualized by Typhoon TRIO imager (GE Healthcare).

### Immunoblots

Cells from ∼90% confluent 6-well plates were trypsinized, washed with phosphate-buffered saline (PBS), resuspended in 100 μl of ELB lysis buffer (250 mM NaCl, 0.1% NP-40, 50 mM 4-(2-hydroxyethyl)-1-piperazineethanesulfonic acid [HEPES], pH 7, 5 mM EDTA) and 25 μl of 5× sample buffer, boiled for 10 min, and stored at −80°C. 30 μg of sample were run on 12% acrylamide gels and transferred to nitrocellulose. Primary and secondary antibodies were diluted in 5% milk in Tris-buffered saline plus 0.1% Tween 20. Primary antibody dilutions were as follows: Aurora B 1:100 (BD Transduction), pAurora A/B/C 1:100 (Cell Signaling), INCENP TSS 1:4000 (a kind gift of Dr. Michael Lampson), α-tubulin 1:250 (DM1a; Sigma-Aldrich), pH3 S10 1:100 (Millipore), α-FLAG 1:10,000 (Sigma A8592).

### Immunofluorescence microscopy

Cells were grown in 12-well plates with 18-mm round coverslips until ∼80% confluent. Coverslips were washed with microtubule stabilizing buffer (MTSB; 100 mM 1,4-piperazinediethanesulfonic acid, pH 6.9, 30% glycerol, 1 mM ethylene glycol tetraacetic acid, and 1 mM MgSO_4_) and fixed with 4% formaldehyde (Tousimis, Rockville, MD) in MTSB at room temperature for 10 min. Coverslips were washed twice with PBS and blocked in triton block (0.2 M glycine, 2.5% FBS, and 0.1% Triton X-100 in PBS) at 4°C. For formaldehyde fixations, cells were pre-extracted with 0.5% Triton X-100 in MTSB for 1–5 min at 37°C. Primary antibody dilutions were as follows: pH3 S10 1:100 (Millipore), pDSN1 S100 1:300 (Welburn et al., 2010), pKNL1 S60 1:1500 (Welburn et al., 2010), INCENP TSS 1:1000 (a kind gift of Dr. Michael Lampson), α-tubulin 1:500 (YL1/2; Biorad), Rhodamine Phalloidin 1:80 (Life Technologies), pDSN1 S109 1:1150 (Welburn et al., 2010). DNA was stained with 2 μg/ml 4′,6-diamidino-2-phenylindole (DAPI). Images were acquired on a Nikon Eclipse Ti-E inverted fluorescence microscope using a CoolSNAP HQ2 or Hamamatsu Orca Flash 4.0 camera and a 100×/1.4 numerical aperture (NA) oil objective. Images are maximum projections of 0.2-μm *z*-stacks deconvolved using the AQI module in Nikon Elements unless otherwise indicated. Quantification was performed in Nikon Elements by measuring the volume of signal over a uniform intensity threshold. The average intensity was then multiplied by the volume and the resulting values were averaged and normalized to control conditions.

Immunofluorescence of methanol fixed *C. elegans* oocytes was performed after gonad extrusion and cryo-preservation. Serial confocal sectioning at 0.2-µm steps in z was conducted to ensure that the entire nucleus was imaged in each oocyte analyzed. To calculate the fluorescence intensity of pH3 in control and GFP-AIR-2 overexpressing animals, the total intensity in a box containing the signal (from a maximum intensity projection) was measured and the camera background was subtracted.

### Statistical analysis

Error bars represent SE unless otherwise specified. Statistical significance was concluded at *p* < 0.05. The Wilcoxon Rank Sum was used to determine significance in quantitative immunofluorescence experiments. The Fisher exact test was used to determine significance in all other experiments unless otherwise noted.

### Biostatistics

All expression data was downloaded from TCGA (BRCA.exp.547. med.txt) and analyzed using custom python scripts, MeV and PRISM. For analysis of AURKB mRNA levels (Figures 7A,C), expression values are expressed as fold median expression across tumors and genes in log2 space. Correlations of expression with AURKB were performed in MeV using Pavlidis Template Matching. p values were then corrected using the Benjamini- Hochberg correction for multiple analyses. For analysis of AURKB correlations with other mRNAs (Figures 7D–F), median-centered expression values as above were expressed as unlogged values. For Figures 7G,H, Peptide data was extracted from cBio to define tumors with high (ratio>1.2) or low (ratio<1.2) AURKB/INCENP ratios. Kaplan Meier curves were generated in PRISM using survival data from the same cBio dataset.

## Data Availability

The original contributions presented in the study are included in the article/Supplementary Material, further inquiries can be directed to the corresponding author.
