# Supplementary material for: Increased Aurora B expression reduces substrate phosphorylation and induces chromosomal instability
Source: Front Cell Dev Biol. 2022 Oct 13;10:1018161. doi: 10.3389/fcell.2022.1018161 (PMC9606593; doi:10.3389/fcell.2022.1018161)
Supplement: Supplementary file 2 [file DataSheet1.pdf]

Supplementary Material

Supplementary Figures

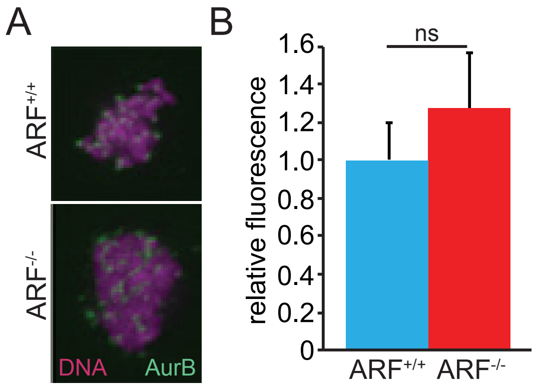

Supplementary Figure 1. Increased expression of Aurora B does not affect Aurora B recruitment to centromeres in ARF$^{-/-}$ MEFs. (A) Quantitative immunofluorescence of Aurora B in ARF$^{+/+}$ and ARF$^{-/-}$ MEFs. (B) Graph showing quantification of Aurora B at centromeres. n > 10 cells from each of 4 independent experiments. ns indicates not significant.

Murine Aurora B

```
SequencingResult      MAQKENAYPWPYGSKTSQSGLNTLSQRVLRKEPATTSALALVNRFNSQSTAAPGQKLAEN      60
NM_011496.2           MAQKENAYPWPYGSKTSQSGLNTLSQRVLRKEPATTSALALVNRFNSQSTAAPGQKLAEN      60
AK132006.1            MAQKENAYPWPYGSKTSQSGLNTLSQRVLRKEPATTSALALVNRFNSQSTAAPGQKLAEN      60
BC003261.1            MAQKENAYPWPYGSKTSQSGLNTLSQRVLRKEPATTSALALVNRSNSQSTAAPGQKLAEN      60
                      ******************************************** ***************

SequencingResult      KSQGSTASQGSQNKQPFTIDNFEIGRPLGKGKFGNVYLAREKKSRFIVALKILFKSQIEK      120
NM_011496.2           KSQGSTASQGSQNKQPFTIDNFEIGRPLGKGKFGNVYLAREKKSRFIVALKILFKSQIEK      120
AK132006.1            KSQGSTASQGSQNKQPFTIDNFEIGRPLGKGKFGNVYLAREKKSRFIVALKILFKSQIEK      120
BC003261.1            KSQGSTASQGSQNKQPFTIDNFEIGRPLGKGKFGNVYLAREKKSRFIVALKILFKSQIEK      120
                      ************************************************************

SequencingResult      EGVEHQLRREIEIQAHLKHPNILQLYNYFYDQQRIYLILEYAPRGELYKELQKSRTFDEQ      180
NM_011496.2           EGVEHQLRREIEIQAHLKHPNILQLYNYFYDQQRIYLILEYAPRGELYKELQKSRTFDEQ      180
AK132006.1            EGVEHQLRREIEIQAHLKHPNILQLYNYFYDQQRIYLILEYAPRGELYKELQKSRTFDEQ      180
BC003261.1            EGVEHQLRREIEIQAHLKHPNILQLYNYFYDQQRIYLILEYAPRGELYKELQKSRTFDEQ      180
                      ************************************************************

SequencingResult      RTATIMEELSDALTYCHKKKVIHRDIKPENLLLGLQGELKIADFGWSVHAPSLRRKTMCG      240
NM_011496.2           RTATIMEELSDALTYCHKKKVIHRDIKPENLLLGLQGELKIADFGWSVHAPSLRRKTMCG      240
AK132006.1            RTATIMEELSDALTYCHKKKVIHRDIKPENLLLGLQGELKIADFGWSVHAPSLRRKTMCG      240
BC003261.1            RTATIMEELSDALTYCHKKKVIHRDIKPENLLLGLQGELKIADFGWSVHAPSLRRKTMCG      240
                      ************************************************************

SequencingResult      TLDYLPPEMIEGRMHNEMVDLWCIGVLCYELMVGNPPFESPSHSETYRRIVKVDLKFPSS      300
NM_011496.2           TLDYLPPEMIEGRMHNEMVDLWCIGVLCYELMVGNPPFESPSHSETYRRIVKVDLKFPSS      300
AK132006.1            TLDYLPPEMIEGRMHNEMVDLWCIGVLCYELMVGNPPFESPSHSETYRRIVKVDLKFPSS      300
BC003261.1            TLDYLPPEMIEGRMHNEMVDLWCIGVLCYELMVGNPPFESPSHSETYRRIVKVDLKFPSS      300
                      ************************************************************

SequencingResult      VPSGAQDLISKLLKHNPWQRLPLAEVAAHPWVRANSRRVLPPSALG                    346
NM_011496.2           VPSGAQDLISKLLKHNPWQRLPLAEVAAHPWVRANSRRVLPPSAL•                    345
AK132006.1            VPSGAQDLISKLLKHNPWQRLPLAEVAAHPWVRANSRRVLPPSAL•                    345
BC003261.1            VPSGAQDLISKLLKHNPWQRLPLAEVAAHPWVRANSRRVLPPSAL•                    345
                      *********************************************
```

Supplementary Figure 2. Sequencing confirmation of mouse Aurora B cDNA. Comparison of the protein sequence of the expressed murine Aurora B cDNA construct to three publicly available murine Aurora B sequences. Two sequences (accession numbers NM_011496.2 and AK132006.1) report a Phe at residue 45 (red), while one sequence (BC003261.1) reports a Ser (blue). The cDNA sequence used here contains Phe at amino acid 45. Note that the cDNA sequence used herein has a glycine in place of the stop codon because of a C-terminal YFP tag.

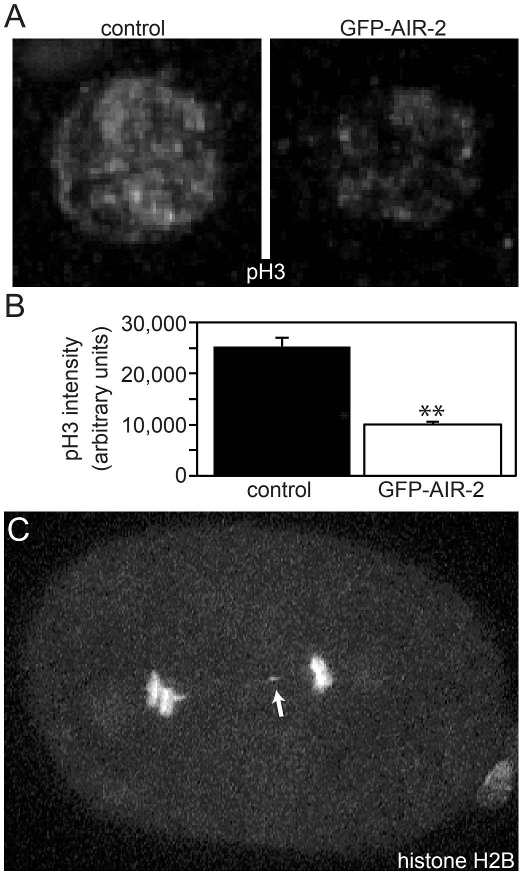

Supplementary Figure 3. Increased expression of the worm homolog of Aurora B, AIR-2, inhibits AIR-2 kinase activity. (A) Images of the Aurora B substrate pH3 in -1 oocytes. (B) Quantification of pH3 shown in A. n=5 control and 5 GFP-AIR-2 oocytes. **=p<0.001. (C) Image of chromosomes during anaphase in a GFP-AIR-2 expressing worm. Arrow denotes a lagging chromosome.

A, *Homo sapiens* Aurora B

```
SequencingResult      MAQKENSYPWPYGRQTAPSGLSTLPQRVLRKEPVTPSALVLMSRSNVQPTAAPGQKVMEN      60
BC080581.1            MAQKENSYPWPYGRQTAPSGLSTLPQRVLRKEPVTPSALVLMSRSNVQPTAAPGQKVMEN      60
BC000442.2            MAQKENSYPWPYGRQTAPSGLSTLPQRVLRKEPVTPSALVLMSRSNVQPTAAPGQKVMEN      60
BC009751.2            MAQKENSYPWPYGRQTAPSGLSTLPQRVLRKEPVTPSALVLMSRSNVQPTAAPGQKVMEN      60
AB519677.1            MAQKENSYPWPYGRQTAPSGLSTLPQRVLRKEPVTPSALVLMSRSNVQPTAAPGQKVMEN      60
                      ************************************************************

SequencingResult      SSGTPDILTRHFTIDDFEIGRPLGKGKFGNVYLAREKKSHFIVALKVLFKSQIEKEGVEH      120
BC080581.1            SSGTPDILTRHFTIDDFEIGRPLGKGKFGNVYLAREKKSHFIVALKVLFKSQIEKEGVEH      120
BC000442.2            SSGTPDILTRHFTIDDFEIGRPLGKGKFGNVYLAREKKSHFIVALKVLFKSQIEKEGVEH      120
BC009751.2            SSGTPDILTRHFTIDDFEIGRPLGKGKFGNVYLAREKKSHFIVALKVLFKSQIEKEGVEH      120
AB519677.1            SSGTPDILTRHFTIDDFEIGRPLGKGKFGNVYLAREKKSHFIVALKVLFKSQIEKEGVEH      120
                      ************************************************************

SequencingResult      QLRREIEIQAHLHHPNILRLYNYFYDRRRIYLILEYAPRGELYKELQKSCTFDEQRTATI      180
BC080581.1            QLRREIEIQAHLHHPNILRLYNYFYDRRRIYLILEYAPRGELYKELQKSCTFDEQRTATI      180
BC000442.2            QLRREIEIQAHLHHPNILRLYNYFYDRRRIYLILEYAPRGELYKELQKSCTFDEQRTATI      180
BC009751.2            QLRREIEIQAHLHHPNILRLYNYFYDRRRIYLILEYAPRGELYKELQKSCTFDEQRTATI      180
AB519677.1            QLRREIEIQAHLHHPNILRLYNYFYDRRRIYLILEYAPRGELYKELQKSCTFDEQRTATI      180
                      ************************************************************

SequencingResult      MEELADALMYCHGKKVIHRDIKPENLLLGLKGELKIADFGWSVHAPSLRRKTMCGTLDYL      240
BC080581.1            MEELADALMYCHGKKVIHRDIKPENLLLGLKGELKIADFGWSVHAPSLRRKTMCGTLDYL      240
BC000442.2            MEELADALMYCHGKKVIHRDIKPENLLLGLKGELKIADFGWSVHAPSLRRKTMCGTLDYL      240
BC009751.2            MEELADALMYCHGKKVIHRDIKPENLLLGLKGELKIADFGWSVHAPSLRRKTMCGTLDYL      240
AB519677.1            MEELADALMYCHGKKVIHRDIKPENLLLGLKGELKIADFGWSVHAPSLRRKTMCGTLDYL      240
                      ************************************************************

SequencingResult      PPEMIEGRMHNEKVDLWCIGVLCYELLVGNPPFESASHNETYRRIVKVDLKFPASVPTGA      300
BC080581.1            PPEMIEGRMHNEKVDLWCIGVLCYELLVGNPPFESASHNETYRRIVKVDLKFPASVPMGA      300
BC000442.2            PPEMIEGRMHNEKVDLWCIGVLCYELLVGNPPFESASHNETYRRIVKVDLKFPASVPMGA      300
BC009751.2            PPEMIEGRMHNEKVDLWCIGVLCYELLVGNPPFESASHNETYRRIVKVDLKFPASVPTGA      300
AB519677.1            PPEMIEGRMHNEKVDLWCIGVLCYELLVGNPPFESASHNETYRRIVKVDLKFPASVPTGA      300
                      ********************************************************* **

SequencingResult      QDLISKLLRHNPSERLPLAQVSAHPWVRANSRRVLPPSALQSVA*                     344
BC080581.1            QDLISKLLRHNPSERLPLAQVSAHPWVRANSRRVLPPSALQSVA*                     344
BC000442.2            QDLISKLLRHNPSERLPLAQVSAHPWVRANSRRVLPPSALQSVA*                     344
BC009751.2            QDLISKLLRHNPSERLPLAQVSAHPWVRANSRRVLPPSALQSVA*                     344
AB519677.1            QDLISKLLRHNPSERLPLAQVSAHPWVRANSRRVLPPSALQSVA*                     344
                      ********************************************
```

B, Alignment of region around human threonine 298

Homo aapiens	291	FPASVP**T**GAQDLI	304
Mus musculus	292	FPSSVP**S**GAQDLI	305
Rattus norvegicus	290	FPSSMP**L**GAKDLI	303
Bos taurus	291	FPPSVP**L**GAQDLI	304
Sus scrofa	291	FPPSVP**A**GAQDLI	304
Xenopus laevis	307	FPPFLS**D**GSKDLI	320
		`**  :  *::***`	

Supplementary Figure 4. Sequencing confirmation of the human Aurora B cDNA. (A) Sequence alignment comparing the human Aurora B protein sequence used here to four 4 publicly available sequences. Two publicly available sequences (accession numbers BC009751.2 and AB519677.1), as well as the sequence used here, contain a Thr at residue 298 (red highlight), while two sequences (BC080581.1 and BC000442.2) contain a Met at this position (blue highlight). Residue 294 is mutated from an alanine to a serine in the sequence published by Tatsuka et al., 1998. (B) Alignment of Aurora B in six different species showing the region around amino acid 298 (bold) in human Aurora B. Note that this residue is not conserved.

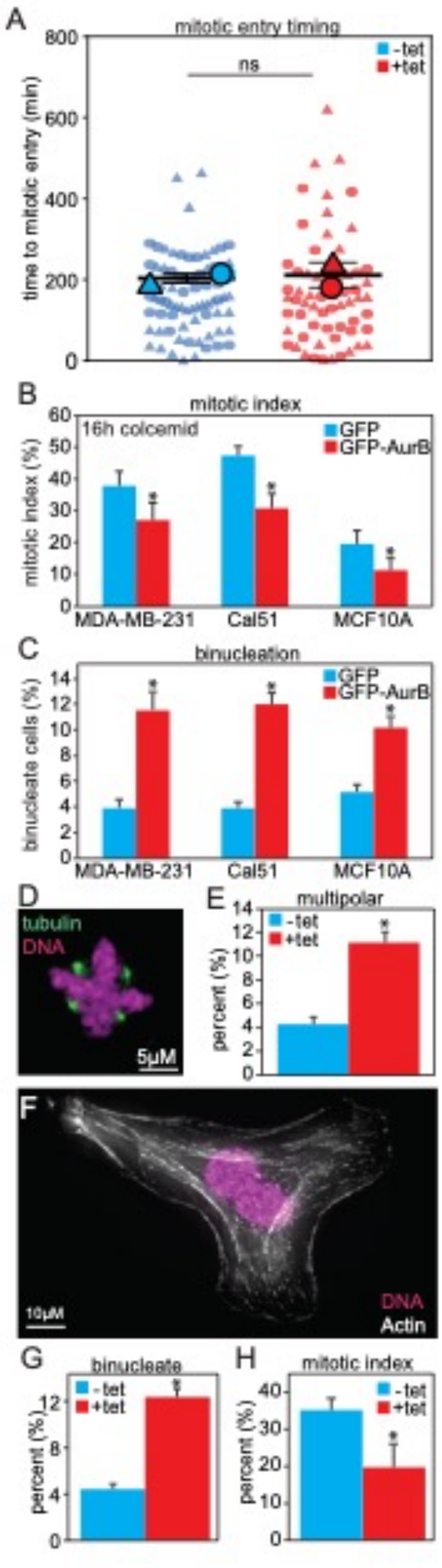

Supplementary Figure 5. Expression of Aurora B in breast cells causes mitotic defects. (A) Tet-inducible expression of untagged Aurora B in MDA-MB-231 cells does not impair mitotic entry in timelapse movies. n > 30 cells from each of two independent experiments. (B-C) Examination of nontransformed MCF10A cells as well as MDA-MB-231 and Cal51 breast cancer cells 48 hours after infection with GFP alone or GFP-Aurora B. (B) Expression of GFP-Aurora B impairs the ability of all three breast cell lines to arrest in mitosis after 16 hours of treatment with the microtubule poison colcemid, indicative of a weakened mitotic checkpoint. n > 250 cells from each of 3 independent experiments. (C) Expression of GFP-Aurora B increases the incidence of binucleation, which is a consequence of cytokinesis failure, in all three breast cell lines. n > 250 interphase cells from each of 3 independent experiments. (D-H) 48 hours of tet-induced expression of untagged Aurora B causes mitotic defects in MDA-MB-231 cells. (D) Representative image of a multipolar spindle. (E) Quantification of multipolar spindles in MDA-MB-231 cells with and without 48 hours of tet-inducible expression of Aurora B. n > 250 mitotic cells from each of 3 independent experiments. (F) Representative image of a binucleate cell. (G) Quantification of binucleation caused by tet-inducible expression of Aurora B. n > 250 interphase cells from each of three independent experiments. (H) Increased expression of Aurora B impairs mitotic checkpoint signaling, resulting in a reduced mitotic index after 16 hours of colcemid treatment. n > 250 cells from each of three independent experiments. * $p<0.05$. ns indicates p value not significant.

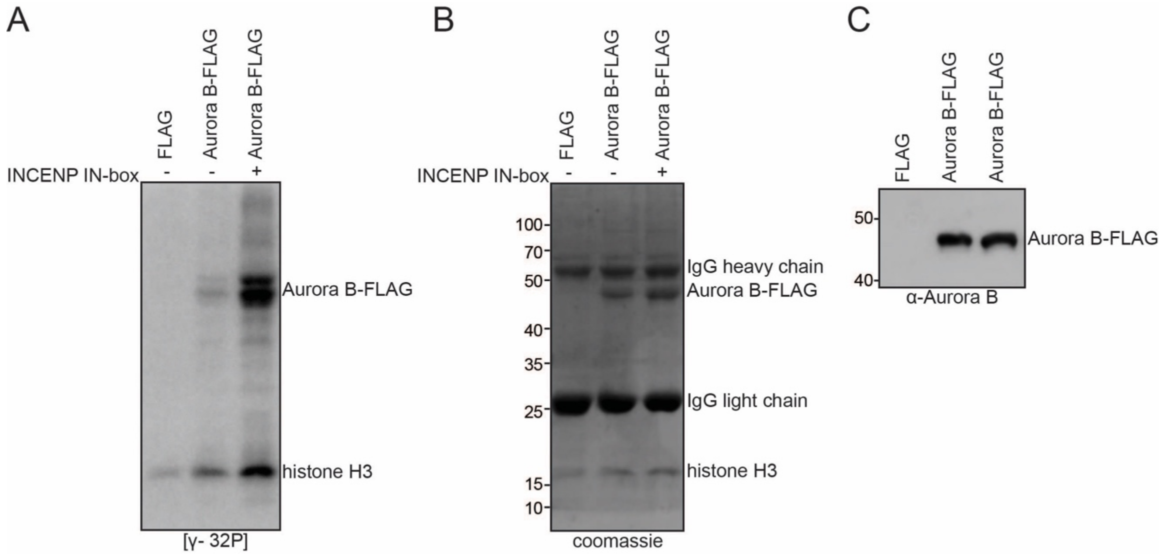

Supplementary Figure 6. Exogenously expressed Aurora B exhibits INCENP-stimulated kinase activity. (A) Kinase assay showing phosphorylation of recombinant histone H3 by Aurora B-3xFLAG that is stimulated by the IN-box of recombinant INCENP. (B) Coomassie showing equal protein loading. (C) Immunoblot of a fraction of the protein/bead mixture after the final wash step prior to the kinase assay showing equivalent immunoprecipitation of Aurora B-3xFLAG prior to addition of kinase assay buffer +/- recombinant INCENP IN-box.

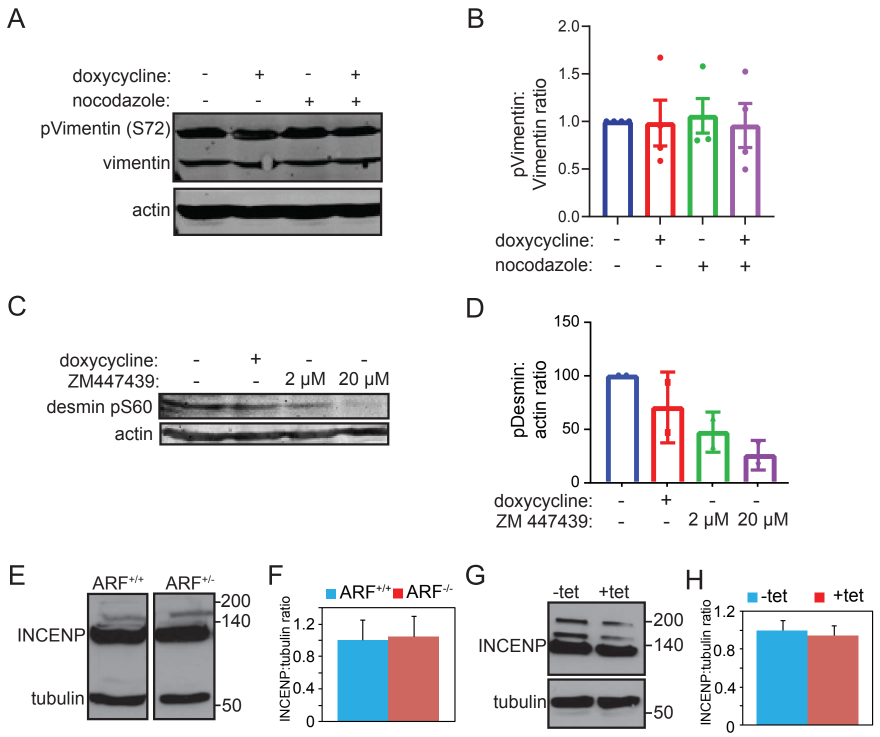

Supplementary Figure 7. Elevated Aurora B expression does not increase cytoplasmic Aurora B kinase activity or alter INCENP protein levels. (A) Immunoblot showing levels of phosphorylated vimentin and total vimentin are similar after doxycycline-induced expression of untagged Aurora B in MDA-MB-231 cells. Where indicated, nocodazole was added for 6 hours. Actin is used as a loading control. (B) Quantification of phosphorylated vimentin (S72) levels in A, normalized to total vimentin. n=4. (C) Immunoblot showing that dox-inducible expression of Aurora B does not increase phospho-desmin (S60) in MDA-MB-231 cells, though this phosphorylation is sensitive to inhibition of Aurora B kinase activity with ZM447439. Cells were treated with colcemid for 4 hours. Actin is used as a loading control. (D) Quantification of pDesmin in C, normalized to actin. n=2. (E) Immunoblot showing ARF$^{-/-}$ primary MEFs, which express increased levels of endogenous Aurora B, have similar levels of INCENP as ARF$^{+/+}$ primary MEFs. Tubulin is used as a loading control. (F) Quantitation of INCENP levels in primary MEFs normalized to tubulin. n=2. (G) Immunoblot showing INCENP levels are unaffected in MDA-MB-231 cells 48 hours after tet addition to induce expression of untagged Aurora B. Tubulin is used as a loading control. (H) Quantitation of INCENP levels in MDA-MB-231 cells that inducibly express untagged Aurora B +/- 48 hours of tet. INCENP expression is normalized to tubulin. n=3.

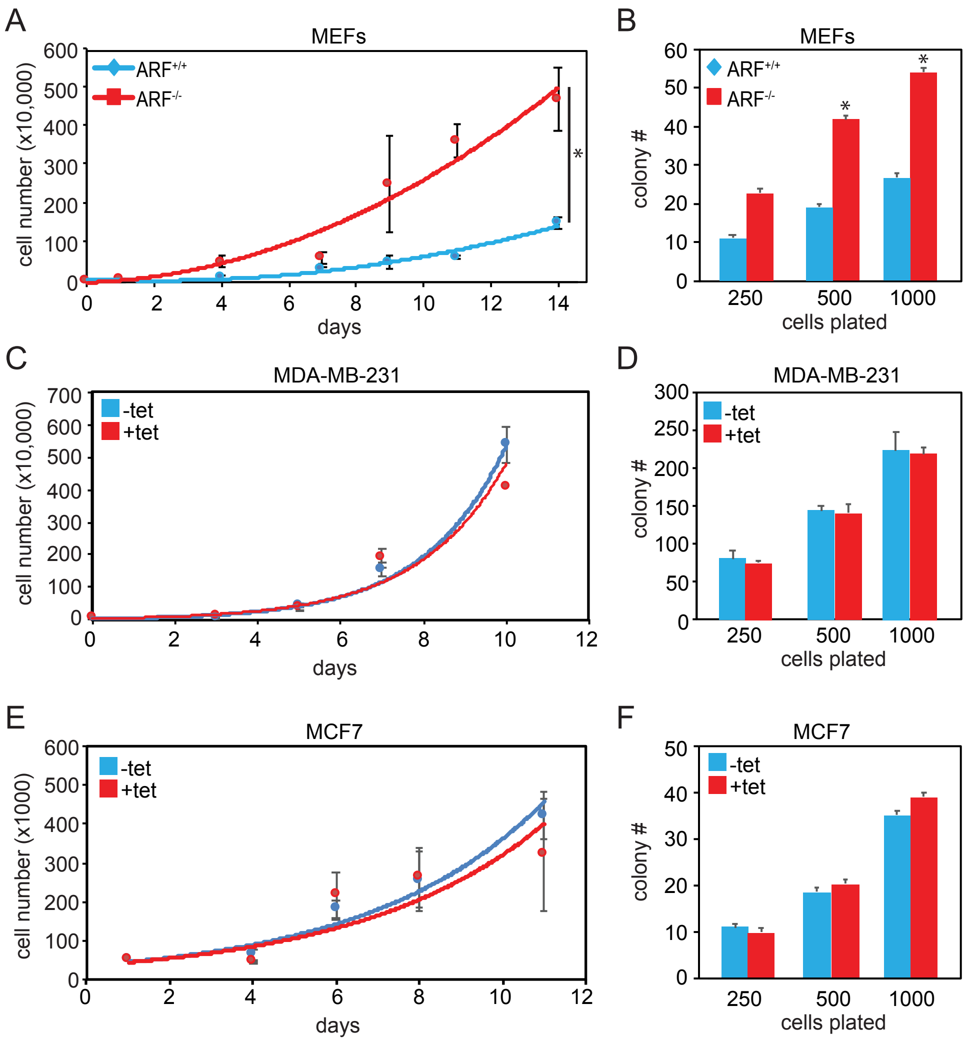

Supplementary Figure 8. Proliferative impact of ARF loss and Aurora B expression. (A-B) ARF loss causes a proliferative advantage in DMSO treated MEFs based on growth curves (A) and colony forming assays (B). (C-D) Tet-inducible expression of untagged Aurora B in MDA-MB-231 cells has no impact on population cell growth (C) or colony forming ability (D). (E-F) Expression of Aurora B-GFP in response to tet in MCF7 cells does not affect proliferation at the population (E) or single cell (F) level. For each panel, n=3 independent experiments. * $p<0.05$.

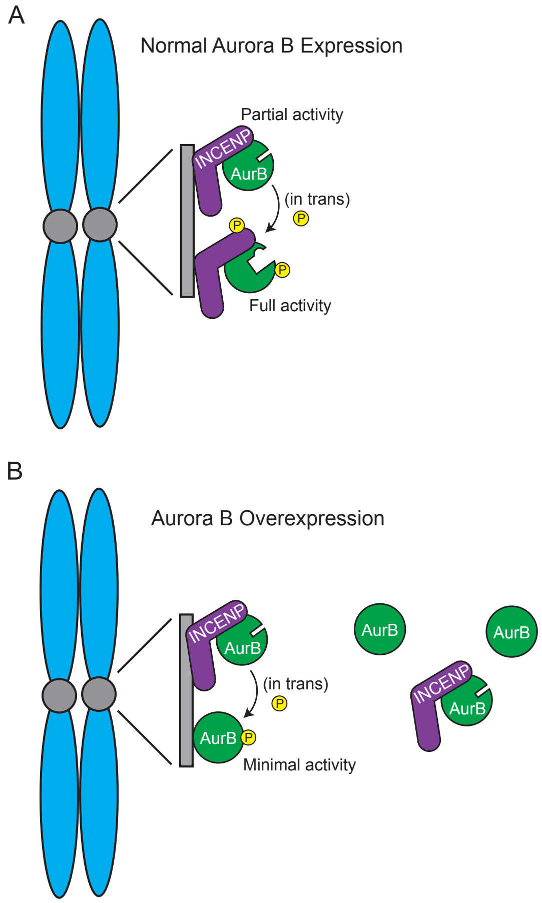

Supplementary Figure 9. Increased Aurora B expression reduces Aurora B kinase activity by titrating INCENP. (A) When expressed at optimal levels, Aurora B and INCENP bind each other at inner centromeres and become partially active. This partial activity is sufficient to phosphorylate other Aurora B:INCENP molecules in trans, which triggers a conformational change and a substantial increase in kinase activity. (B) When Aurora B expression is increased, excess cytoplasmic Aurora B titrates INCENP from the inner centromere to the cytoplasm. Although partially active Aurora B:INCENP complexes can phosphorylate Aurora B monomers in trans at inner centromeres, the phosphorylated Aurora B monomers are only minimally active. The decrease in the local concentration of Aurora B:INCENP complexes at inner centromeres results in decreased Aurora B kinase activity.
